# The affective priming paradigm as an indirect measure of food attitudes and related choice behaviour

**DOI:** 10.3758/s13423-020-01764-1

**Published:** 2020-06-30

**Authors:** Loukia Tzavella, Leah Maizey, Andrew D. Lawrence, Christopher D. Chambers

**Affiliations:** CUBRIC, School of Psychology, Cardiff University, Cardiff, UK

**Keywords:** Affective priming, Foods, Liking, Choice, Healthiness

## Abstract

In this Registered Report, we assessed the utility of the affective priming paradigm (APP) as an indirect measure of food attitudes and related choice behaviour in two separate cohorts. Participants undertook a speeded evaluative categorization task in which target words were preceded by food primes that differed in terms of affective congruence with the target, explicit liking (most liked or least liked), and healthiness (healthy or unhealthy). Non-food priming effects were tested as a manipulation check, and the relationship between food priming effects and impulsive choice behaviour was also investigated using a binary food choice task. As predicted, priming effects were observed for both healthy and unhealthy foods, but there was no difference in the magnitude of these effects. This may suggest that the paradigm is most sensitive to affective, but not cognitive, components of attitudes (i.e., healthiness), but alternative theoretical explanations and implications of this finding are discussed. Food and non-food priming effects were observed in both reaction time (RT) and error rate (ER) data, but contrary to expectations, we found no association between food RT priming effects and choice behaviour. All findings from confirmatory analyses regarding RT and ER priming effects, and the absence of the expected correlations between priming effects and impulsive food choices, were successfully replicated in the online cohort of participants. Overall, this study confirms the robustness of the APP as an indirect measure of food liking and raises questions about its applied value for research of eating behaviours.

**Electronic supplementary material:**

The online version of this article (10.3758/s13423-020-01764-1) contains supplementary material, which is available to authorized users.

There is an emerging need for a greater understanding of attitudes towards foods that may drive unhealthy eating behaviours, such as overeating. Attitudes reflect “object-evaluation associations” that can be retrieved from memory and influence behaviour towards the attitude object (Klauer & Musch, 2003). For example, individuals may respond positively to a food that contains intrinsically rewarding ingredients (e.g., sugar, fat), with the positive evaluation automatically activated by the learned association between reward and consumption. Evaluations of foods arise from both affective and cognitive components of attitudes (Marty et al., 2017). The affective component reflects an individual’s hedonic reaction to the sensory properties of foods, commonly referred to as food liking, which is a central determinant of dietary choice (Eertmans, Baeyens, & Van den Bergh, 2001). The cognitive component may involve thoughts about the nutritional value of a food item and potential health consequences (Trendel & Werle, 2015). This study examined the methodological validity of an indirect measure of attitudes—the affective priming paradigm (APP; Fazio & Olson, 2003; Fazio, Sanbonmatsu, Powell, & Kardes, 1986; Hermans, De Houwer, & Eelen, 2001; Klauer & Musch, 2003)—and the extent to which priming measures were sensitive to affective (i.e., liking) and cognitive (i.e., healthiness) components of food attitudes. The association between priming measures and food-choice behaviour was also investigated.

The interplay between affective and cognitive components of attitudes may be paramount to the understanding of eating behaviours, including food choices. Appetitive foods and their cues, such as sight or smell, can induce positive affective reactions (Blechert, Meule, Busch, & Ohla, 2014) and activate the brain’s reward circuits associated with “wanting” and “liking” (Berridge, Ho, Richard, & DiFeliceantonio, 2010). In food-rich societies, where high-calorie foods are heavily promoted, such cue-evoked positive reactions are frequent and can drive impulsive food choices (Zoltak, Veling, Chen, & Holland, 2018) that likely contribute to overeating and other unhealthy eating behaviours (Berridge et al., 2010; Lawrence, Hinton, Parkinson, & Lawrence, 2012; Sato, Sawada, Kubota, Toichi, & Fushiki, 2016). These impulsive food choices are not guided by deliberate processes, such as the consideration of consequences (Veling et al., 2017). Cognitive components of attitudes include social norms and individual beliefs about the attitude object (i.e., foods), such as nutrition and health consequences, and should be considered as determinants of eating behaviours (Eertmans et al., 2001). Interestingly, cognitive and affective components of attitudes can interact, as implicit measures can be influenced by various sources of valence, such as caloric content, economic cost, and effects on one’s health (Verhulst, Hermans, Baeyens, Spruyt, & Eelen, 2006). For example, unhealthy foods can be perceived to be tastier than healthy foods and chosen for consumption more frequently, even if individuals are not consciously aware of the association between healthiness and tastiness (Ackermann & Palmer, 2014).

The APP has been previously applied to the food domain as an implicit, or indirect, measure of attitudes (e.g., Lamote, Hermans, Baeyens, & Eelen, 2004; Roefs, Herman, MacLeod, Smulders, & Jansen, 2005a). The current study employed a variant of the APP where attitude objects are presented as primes and are unrelated to the primary task of identifying the evaluative connotation of target words presented after the primes (Fazio & Olson, 2003). Participants were asked to perform an evaluative categorization task, identifying target words as either positive or negative when preceded by either most liked (i.e., positive) or least liked (i.e., negative) food primes (see Figs. 1 and 2). Here, the main outcome of interest is the affective priming effect, which manifests as faster responses (and/or lower error rates) on affectively congruent (i.e., most liked food-positive target or least liked food-negative target) than incongruent trials (i.e., most liked food-negative target or least liked food-positive target). In contrast to other indirect measures of (food) attitudes, such the implicit association test (Greenwald, McGhee, & Schwartz, 1998), this APP task variant does not require an evaluative response towards the prime, and participants are explicitly instructed to not pay attention to the primes (pictures or words). Affective priming effects can be explained by response competition/facilitation processes (Fazio & Olson, 2003; Wentura & Degner, 2010; but see Discussion section), and in the food domain they are often utilized as a measure of liking or preferences. We posit that such priming measures may be influenced by both affective and cognitive components of attitudes, and their association with food-choice behaviour should be examined further.

**Fig. 1:**
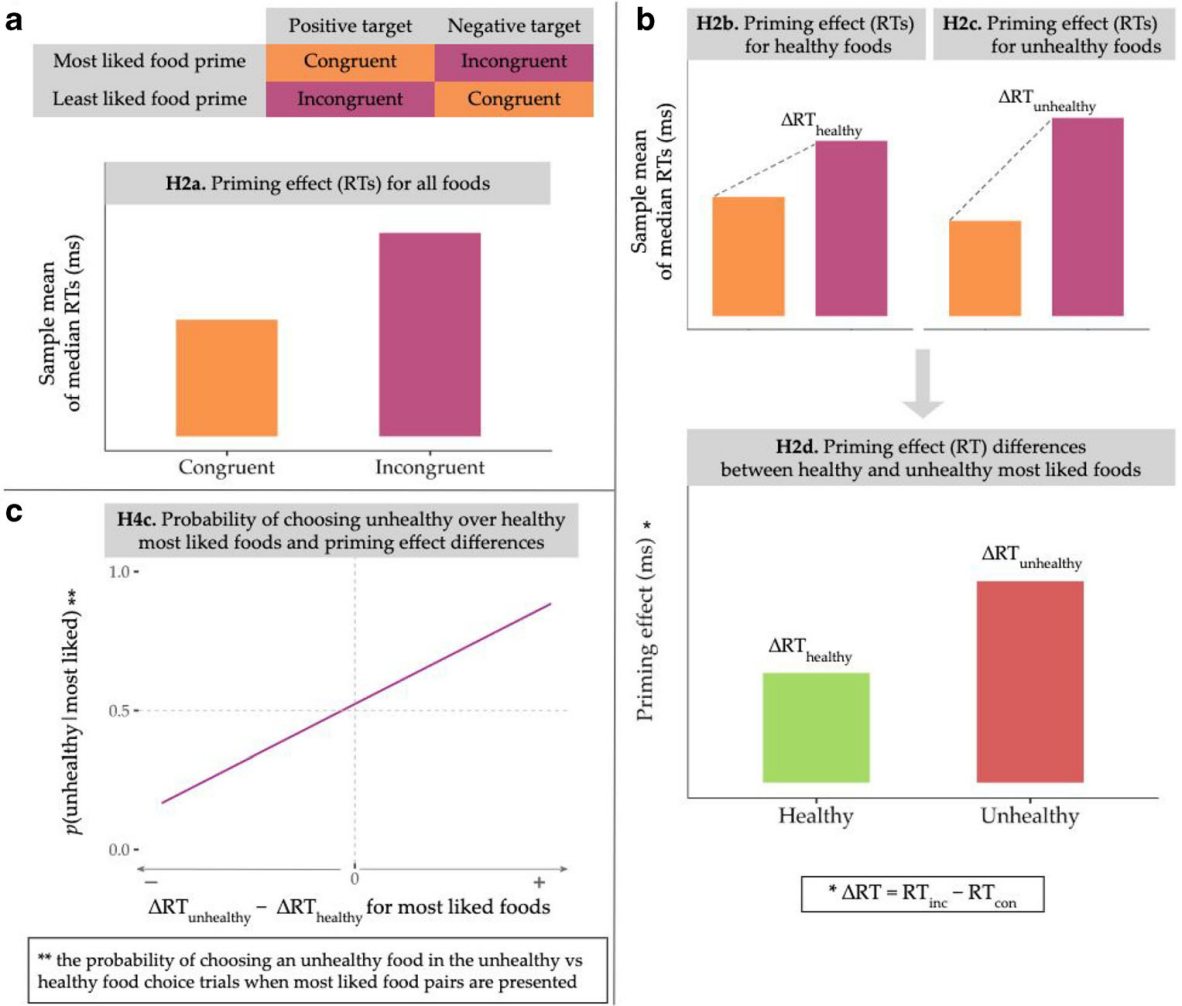
Schematic of affective priming paradigm contrasts and selected hypotheses. **a** Congruence in the affective priming paradigm is defined by the prime–target pairs. The trial is classified as *congruent* when a most liked food prime is paired with a positive target, and *incongruent* when paired with a negative target. Additionally, the trial is *congruent* when a least liked food prime is paired with a negative target, and *incongruent* when paired with a positive target. A priming effect for all foods (H2a) would be shown by faster sample means of median RTs (ms) in congruent versus incongruent trials (RT_con_ < RT_inc_). (Details for all RT calculations can be found in the Measures and Indices section.) Priming effects for RTs (H2) are shown here only for demonstration purposes, but the priming effects in terms of ERs are in the same direction (ER_con_ < ER_inc_; see H3 predictions). **b** Priming effects were expected for both healthy (H2b) and unhealthy food primes (H2c). It was also hypothesized that the priming effect would be greater for unhealthy than for healthy food primes (H2d). The RT priming effect was calculated as the difference in median RTs for incongruent and congruent trials (medianRT_inc_ − medianRT_con_) at the participant level, and the sample means of these difference scores were then compared across conditions (healthy vs. unhealthy). **c** The probability of choosing unhealthy over healthy most liked foods in the unhealthy versus healthy food-choice task trials was hypothesized to positively correlate (linearly) with individual differences in RT priming effects between unhealthy (∆RT_unhealthy_) and healthy (∆RT_healthy_) most liked food prime trials (H4c). The latter was examined using difference scores (∆RT_unhealthy_ − ∆RT_healthy_) in which a positive value indicates that participants had a larger priming effect for unhealthy most liked food primes. H4a and H4b are not shown here, but also posit expected positive linear correlations between variables. *Note*. Hypotheses graphs are not based on actual or simulated data and are for illustrative purposes only. RT = reaction time; RT_con_ = RTs in congruent trials; RT_inc_ = RTs in incongruent trials; ER = error rate; ∆RT = RT difference score (as shown in the formulas)

**Fig. 2:**
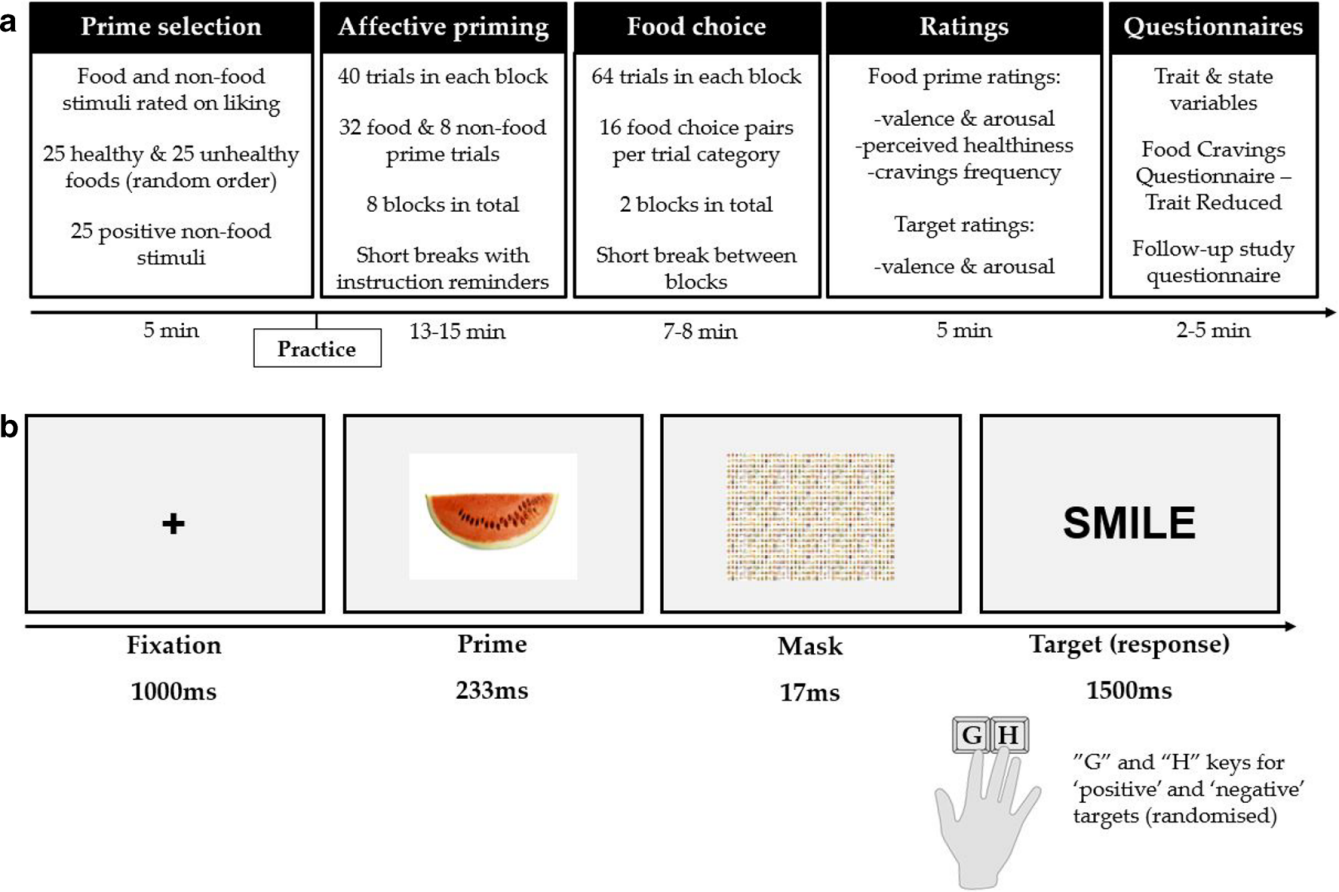
Schematic of the study procedure and affective priming paradigm. **a** Primes for the affective priming paradigm (APP) were selected based on participants’ liking ratings, with the subsequent APP consisting of eight blocks, including 32 food and eight non-food prime trials per block. The food-choice task followed the APP and included two blocks of 64 trials. Participants then rated all primes, and targets and were presented with three short questionnaires in the depicted order. **b** The APP involved an evaluative categorization task, where participants categorized target words as positive or negative as quickly and accurately as possible. After a central fixation cross (1,000 ms), a prime (food or non-food) was presented for 233 ms, followed by a mask. Participants must respond within 1,500 ms of target onset using the “G” and “H” keys for “positive” and “negative” (randomized across participants) using their index and middle fingers. Finger placement on the assigned keys depended on the participant’s dominant hand

The APP has been shown to capture the evaluation of foods (i.e., liking) through observed priming effects for both reaction times and error rates (Lamote et al., 2004), even when attitudes were only recently acquired in laboratory settings (Verhulst et al., 2006). Although the affective component of food attitudes has been successfully investigated using the APP, previous studies have yielded mixed evidence for its utility in identifying the influence of cognitive components, such as health-related values, on implicitly measured food attitudes. While some studies have found that healthiness or fat content may have no influence on the affective priming effect (Becker, Jostmann, Wiers, & Holland, 2015; Roefs, Herman, et al., 2005a), other evidence suggests that priming can reflect preference for low-fat over high-fat palatable foods, potentially attributed to health concerns (Roefs, Stapert, et al., 2005b).

Overall, there has been moderate evidence to suggest that the APP can tap into the affective components of foods. This study aimed to address three questions that are central to establishing the methodological utility of the APP in eating behaviour. First, can priming effects be obtained for most liked and least liked foods, as expected by previous findings? Second, is this paradigm sensitive to cognitive components of attitudes, such as the healthiness of the foods? Finally, are priming effects for foods that vary in liking and healthiness associated with impulsive choices to consume these foods?

## Hypotheses

The study tested several confirmatory hypotheses regarding the utility of the APP as an indirect measure of food attitudes. Priming effects were examined using both median reaction times for correct responses (RTs) and error rates (ERs). The relationship between priming measures and impulsive food choices were also investigated. A schematic diagram of the APP contrasts and selected hypotheses is shown in Fig. 1. In the Preregistered Analyses section, statistical tests for four categories of predictions (H1–H4) are outlined. These were the exclusive set of a priori hypotheses. For confirmatory analyses, all hypotheses were tested and reported with no changes to the specified independent variables, dependent variables, or any other variables, variable derivations, stated statistical transformations, or data exclusions within each test. The hypotheses, analyses, manipulated, and nonmanipulated variables, and measurements in the Methods and Analyses sections were therefore complete, necessary, immutable, and exclusive for all preregistered confirmatory outcomes.

H1. Positive priming effect for non-food primes as a manipulation check for the APP^1^

H1a. RTs would be, on average, faster in congruent than in incongruent non-food prime trials.

H1b. ERs would be, on average, lower in congruent than in incongruent non-food prime trials.

H2. Priming effects (RTs) for healthy and unhealthy foods

H2a. RTs would be, on average, faster in congruent than in incongruent food prime trials.

H2b. RTs would be, on average, faster in congruent than in incongruent *healthy* food prime trials, specifically.

H2c. RTs would be, on average, faster in congruent than in incongruent *unhealthy* food prime trials, specifically.

H2d. The priming effect (RT difference scores) would be, on average, greater for unhealthy than for healthy most liked food prime trials (see Measures and Indices section for priming effect calculation).

H3. Priming effects (ERs) for healthy and unhealthy foods

H3a. ERs would be, on average, lower in congruent than in incongruent food prime trials.

H3b. ERs would be, on average, lower in congruent than in incongruent *healthy* food prime trials, specifically.

H3c. ERs would be, on average, lower in congruent than in incongruent *unhealthy* food prime trials, specifically.

H3d. The priming effect (ER difference scores) would, be on average, greater for unhealthy than for healthy most liked food prime trials.

H4. Relationship between food choices and observed priming effects (RTs)

H4a. The probability of choosing a most liked over a least liked food from within a pair of healthy food stimuli would positively correlate with the priming effect (RTs) in healthy food prime trials.

H4b. The probability of choosing a most liked over a least liked food from within a pair of unhealthy food stimuli would positively correlate with the priming effect (RTs) in unhealthy food prime trials.

H4c. The probability of choosing an unhealthy over a healthy most liked food would positively correlate with the difference in priming effects (RTs) between unhealthy and healthy most liked food prime trials.

Preregistered hypotheses for priming effects were proposed for both speed (RT) and accuracy (ER) measures. In response priming procedures without strict time windows (e.g., 300–450 ms) priming effects are most commonly observed in RTs (Wentura & Degner, 2010), but we assume that such effects may be observed in either speed and/or accuracy performance (RT_con_ < RT_inc_ and/or ER_con_ < ER_inc_). In addition, accuracy data should be inspected for potential speed–accuracy trade-offs. For example, participants could purposefully delay their responses on incongruent trials to improve accuracy, producing a priming effect for RTs, but a reverse effect for error rates (i.e., ER_con_ > ER_inc_). Therefore, support for observed priming effects would be dependent on both speed and accuracy hypotheses, as shown in the expression^2^ below, where there should be no effects in the opposite direction (RT_con_ > RT_inc_ or ER_con_ > ER_inc_), and there should be evidence for either RT or ER priming effects (RT_con_ < RT_inc_ or ER_con_ < ER_inc_). A contingent analysis plan for testing these hypotheses (i.e., follow-up tests) when the effects were not in the expected direction was preregistered (see Preregistered Analyses).

$$ \left\{\neg \left[\left({\mathrm{RT}}_{\mathrm{con}}>{\mathrm{RT}}_{\mathrm{inc}}\right)\bigvee \left({\mathrm{ER}}_{\mathrm{con}}>{\mathrm{ER}}_{\mathrm{inc}}\right)\right]\right\}\bigwedge \left[\left({\mathrm{RT}}_{\mathrm{con}}<{\mathrm{RT}}_{\mathrm{inc}}\right)\bigvee \left({\mathrm{ER}}_{\mathrm{con}}<{\mathrm{ER}}_{\mathrm{inc}}\right)\right] $$

## Methods

### Data collection protocol

#### Study setting

The study was undertaken in both laboratory (group testing) and online settings using Inquisit 5 (http://www.millisecond.com). The study protocol was matched for the two collected data sets, which were analyzed and reported separately. The primary data set stemmed from the laboratory setting, as this would allow us to examine consequential food choices (see Food-Choice Task section). The online data set would directly replicate any findings on the APP as an indirect measure of food attitudes (H1–H3) and examine whether priming measures were associated with nonconsequential food choices (i.e., choices are not motivated by the offer of real food at the end of the experiment). This data collection protocol would also provide insights into data quality and potential differences in the utility of the APP between laboratory and online settings (see Data Quality Checks in Supplementary Material).

#### Sampling plan and participants

A sequential Bayes factor (SBF) design (Schönbrodt, Wagenmakers, Zehetleitner, & Perugini, 2017) was employed with a predefined minimum sample size (*n*_min_ = 40) and a maximum number of participants (*n*_max_ = 200) for each study setting (laboratory and online). A threshold of BF_10_ ≥ 10 would indicate *strong* evidence for the alternative hypothesis (H1) compared with the null (H0), whereas a threshold of BF_01_ ≥ 10 would correspond to *strong* evidence for H0 relative to H1 (see Lee & Wagenmakers, 2013). For every 10 participants collected, data were inspected for potential exclusions (see Data Exclusions section), and interim analyses were conducted to check whether these evidential thresholds were met for *all* confirmatory hypotheses. If not, data from another 10 participants were collected, and this process was repeated until either the desired level of evidence for all confirmatory hypotheses was obtained, or *n*_max_ had been reached.

Although frequentist power analysis was not appropriate for an SBF design, a Bayes factor design analysis (BFDA; see Fig. S1 in the Supplementary Material) was conducted to assess the probability of the proposed design generating misleading evidence (Schönbrodt & Wagenmakers, 2018). Analyses were performed for all preregistered hypotheses, as in directional *t* tests for priming-related hypotheses (H1–H3) and directional correlations for food-choice task predictions (H4). The design priors were consistent with the analysis priors that would be employed for Bayesian *t* tests and correlations (see Preregistered Analyses section). Only the BFDA results were considered for the design of the study, and no other power analyses were performed.

Recruitment was conducted via advertisements at Cardiff University and Prolific^3^ (https://www.prolific.ac/), and data were collected in both laboratory and online settings (see Study Setting section). We recruited 254 individuals via Prolific, and excluding 18 recorded drop-outs, 30 recruited individuals were not eligible to participate and quit the study (see Fig. 4). In laboratory settings, a total of 205 participants were recruited. When the maximum number of participants was reached for APP analyses (H1–H3), we had to recruit additional participants to also reach *n*_max_ for H4 because of different data exclusion criteria (see Sample Characteristics section; Fig. 4). A total of 134 participants recruited via the Experimental Management System (EMS) received course credits when eligible (e.g., undergraduate students), and 71 participants not eligible for course credits received monetary reimbursement (£6). Participants performing the study via Prolific were rewarded £4.50 upon completion.^4^

The complete and exhaustive set of inclusion and exclusion criteria for participation in the study were as follows. Eligible participants were at least 18 years of age, had normal or corrected-to-normal vision, including normal colour vision, and spoke English as their first or second language. Exclusion criteria included being on a diet and/or have recently been taking diet pills, a past and/or current history of eating disorders and food allergies and/or intolerances. Screening survey questions can be found at https://osf.io/n36cg, and all criteria were based on self-report. Further post hoc exclusions of participants from preregistered analyses are presented in the Data Exclusions section.

The study was approved by the local Research Ethics Committee at the School of Psychology, Cardiff University. All eligible participants provided informed consent and were debriefed. The study employed a within-subjects design, and blinding of participants and/or experimenters was not applicable. However, participants were not made aware of the study aims before completion. Also, the contact between the experimenter and participants was minimized as data was collected online and in group laboratory settings.

### Procedure

Recruited participants confirmed their eligibility and proceeded to provide their consent and choose their study setting (laboratory or online). Participants also indicated their dominant, or preferred, hand for performing the study tasks. A schematic of the study procedure is shown in Fig. 2. The prime selection process required participants to complete a rating task where they rated how much they like food and non-food stimuli (see Prime Selection section). Participants completed a short APP practice block (16 trials), where they received feedback on both the speed and accuracy of their responses. Participants completed eight blocks of the task in total, with short breaks in between and instruction reminders.

After the APP, participants performed a food-choice task (FCT; see Food-Choice Task section), consisting of two blocks in total. In laboratory settings, participants received a food item chosen during the task at the end of the study. In online settings, food choice was not consequential in terms of real consumption. Ratings for all primes and targets (see Prime and Target Ratings section) were provided after the FCT for exploratory analyses. Participants were presented with three short questionnaires^5^ (see Questionnaires section). The total duration of the study per participant was 40–50 minutes, after which participants were debriefed.

### Affective priming paradigm

#### Prime selection

The food primes were selected from 25 healthy and 25 unhealthy foods that were rated on liking, as measured using a visual analogue scale ranging from −100 (*strongly dislike*) to 100 (*strongly like*). Four unhealthy and four healthy foods that had the maximum rating were selected as “most liked” primes, and four unhealthy and healthy foods that had the minimum rating were chosen as “least liked” primes. For each selected food category (e.g., apples for healthy most liked), there were two exemplars in the APP. Instructions highlighted that “the rating task includes foods that could be either liked or disliked” to minimize the potential of social desirability bias whereby participants consistently rate foods on the positive end of the scale. Non-food primes were selected from 25 positive images from various categories, such as animals, that comprised several items (e.g., kitten, puppy, panda). The food ratings were always presented first, and the order of healthy and unhealthy food rating blocks was randomized across participants. Foods in each block were presented in a random order. More details about the food and non-food stimuli can be found in the Supplementary Material.

#### Task design

The APP involved an evaluative categorization task (see Fig. 2) in which participants categorized target words as either positive or negative. The targets were preceded by either “positive” or “negative” food primes, as well as positive non-food primes (manipulation check). The food prime trials involved a 2 × 2 × 2 design, with the manipulated variables of healthiness (healthy vs. unhealthy), affective congruence (congruent vs. incongruent), and liking (most liked vs. least liked). Non-food prime trials differed only in affective congruence. Each block of 40 trials consisted of 16 healthy and 16 unhealthy food prime trials as well as 8 non-food prime trials. Congruent and incongruent prime–target pairs appeared with equal probability for all trials. There were 32 positive and 32 negative targets in total (see Supplementary Material), which appeared randomly with equal probability across two consecutive blocks for food prime trials. Targets for non-food prime trials were presented randomly across eight blocks.^6^

Participants were instructed to categorize the words as quickly and as accurately as possible. Participants responded using the “G” and “H” keys, as explained in Fig. 2. Each trial commenced with a central fixation cross followed after 1,000 ms by the prime. Following a 233-ms interstimulus interval (ISI), the prime was succeeded by a backward mask (17 ms) to limit subjective awareness of the primes, constructed from a mosaic of various food stimuli with different colour compositions (Wentura & Degner, 2010). The stimulus-onset asynchrony (SOA) between prime and target was 250 ms. The response window begun on target onset (i.e., 1,250 ms), and participants had a maximum reaction time of 1,500 ms. Each trial ended either when a response was registered or when the maximum total trial duration was reached (2,750 ms). A trial was considered incorrect if the target categorization was wrong or participants did not respond within 1,500 ms. All stimuli were presented centrally, and pictures had their relative dimensions set to 40% of the vertical and horizontal width of the presentation window. The targets and fixation cross (+) were presented in black, bold Arial fonts. Words were presented in uppercase letters against a uniform grey background.

### Food-choice task

The FCT involved binary food choices, adapted from previous literature (Veling et al., 2017; Zoltak et al., 2018). Participants were instructed to choose the foods that they would prefer to eat at the end of the experiment. To measure consequential food choices, in laboratory settings participants were instructed that one of their choices would be selected by the researcher(s) and they would be given the food item they had chosen on that occasion. The selection of the food was not random due to the unsuitability of certain foods for laboratory storage (e.g., fast decay of fruits). The proposed selection process was in line with instructions used in previous literature (Veling et al., 2017). The researcher(s) selected an item from the list of suitable foods and restricted selection to foods rated as “most liked” by the participants (see Supplementary Material). In online settings, participants did not receive a food item at the end of the study, and thus choices were not consequential. In the laboratory, we also provided participants with bottled water after screening to minimize the potential impact of thirst levels on food choices.

**Fig. 3:**
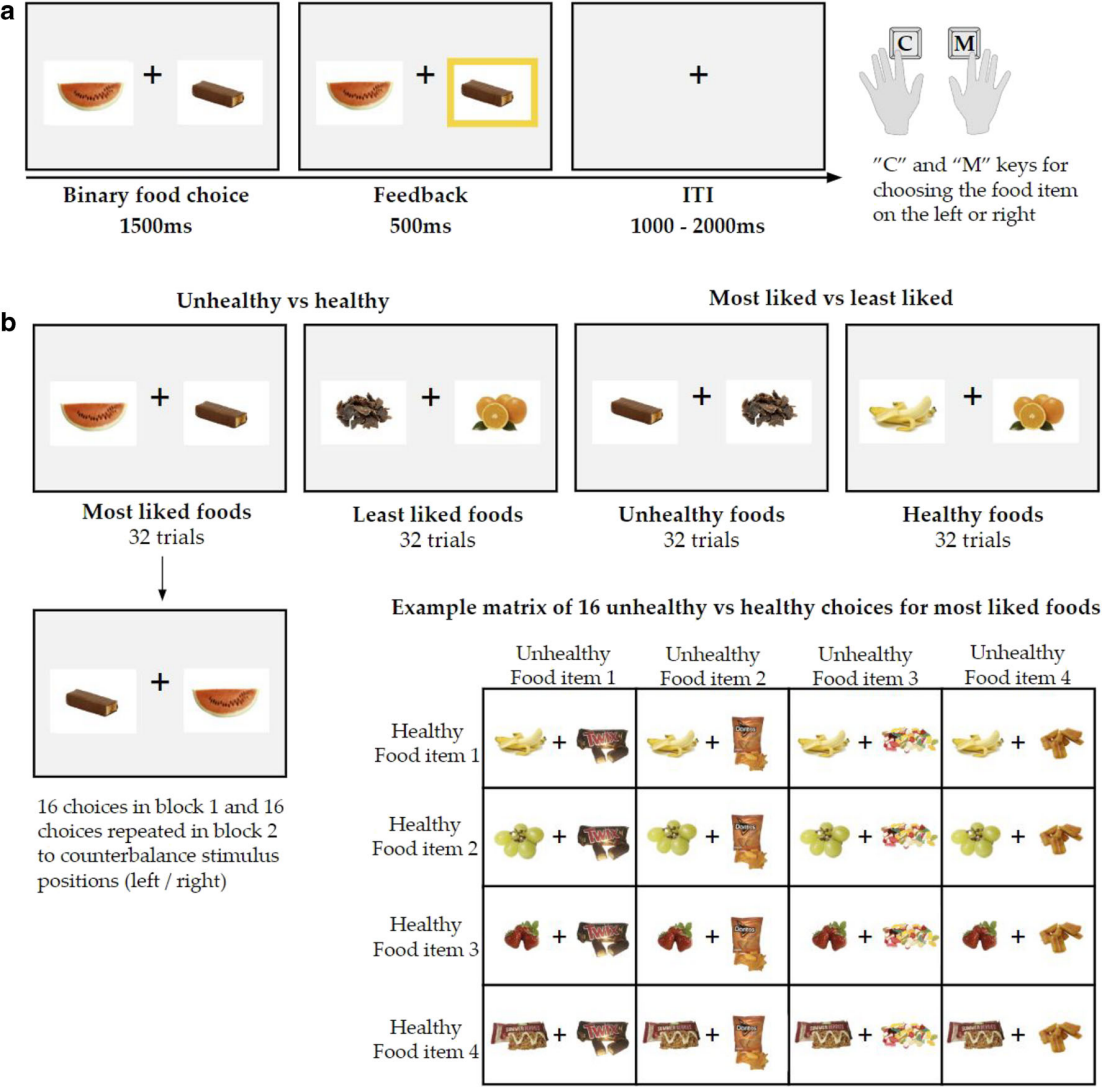
Schematic of the food-choice task and different trial types. **a** Participants made binary food choices within 1,500 ms between two food items presented on the left and right of a central fixation cross. Their response was followed by visual feedback (500 ms). The intertrial interval (ITI) varied randomly (1,000–2,000 ms). **b** The two main trial types involved unhealthy versus healthy and most liked versus least liked choices. There were two categories of food pairs for each trial type. In *unhealthy versus healthy* trials (*N* = 32 per block), participants chose between most liked foods (*N* = 16) or least liked foods (*N* = 16), as shown in the example matrix. In *most liked versus least liked* trials, participants chose between unhealthy (*N* = 16) or healthy (*N* = 16) foods. There were two blocks in total, and choices were repeated in Block 2 to counterbalance stimulus positions, as shown above

Each trial in the FCT (see Fig. 3a) involved the simultaneous presentation of two food items on the left and right of a central fixation cross, which participants would choose between using the “C” and “M” keys.^7^ A response had to be registered within a maximum of 1,500 ms, and participants would then be presented with response feedback (500 ms) where their confirmed choice would be highlighted (i.e., a yellow frame around the selected food). A central fixation cross was presented during the intertrial interval (1,000–2,000 ms^8^).

Participants were instructed to make their choices quickly, and time pressure would help ensure that food choices were not deliberate, reducing the probability of demand characteristics (Veling et al., 2017). Feedback was presented if participants did not respond within 1,500 ms, instructing them to choose faster (“Please try to choose faster”—1,000 ms). To avoid loss of data, missed trials were repeated, and only one repetition per trial was allowed. For each design cell of the APP (healthiness × liking) there were four food categories included in the FCT. All food prime categories were included in the FCT and represented by the primary exemplars (i.e., stimuli used in prime selection). Two main types of trials were presented, and each type had two categories (see Fig. 3b). The FCT comprised 128 binary choices in total and was split into two blocks of 64 trials with a short intervening break.

### Prime and target ratings

Participants explicitly evaluated all prime categories and targets for exploratory analyses. Food primes were evaluated for valence, arousal, perceived healthiness, and frequency of cravings. Non-food primes were also evaluated for valence and arousal. Ratings were only obtained for the primary exemplars. All targets were evaluated for valence and arousal (see https://osf.io/n36cg).

### Questionnaires

#### Trait and state variables

An initial questionnaire recorded several trait and state variables that could be associated with eating behaviours and related information (available at https://osf.io/n36cg). These variables included how long ago participants had their last meal, whether they followed a specific diet, and hunger levels. Self-reported height and weight was recorded to calculate the participants’ body mass index (BMI: kg/m^2^). Participants also indicated their gender and ethnicity (optional). Participants then proceeded to complete the short version of the Food Cravings Questionnaire—Trait-reduced (FCQ-T-r; Meule, Hermann, & Kübler, 2014). FCQ-T-r consists of 15 items scored on a 5-point scale (*strongly disagree* to *strongly agree*).

#### Follow-up study questionnaire

At the end of the study, participants completed a follow-up study questionnaire (see Supplementary Material), where they were asked to answer questions about their performance in the APP (e.g., response strategies). Participants also indicated the number of occasions they were interrupted during the word task (see Waters & Li, 2008). The survey included an instructional manipulation check to examine whether participants were paying attention to the instructions as well as a questionnaire attention check measure (Kees, Berry, Burton, & Sheehan, 2017). Participants’ performance on the data quality assurance measures would later be compared for online and laboratory settings in exploratory analyses (see Supplementary Material).

## Analyses

### Measures and indices

All planned comparisons are outlined in the section below, where RT_con_ and RT_inc_ denote the sample means of individual median correct RTs in congruent and incongruent trials, and ER_con_ and ER_inc_ refer to the mean error rates in congruent and incongruent trials, respectively. At the level of participants, median RTs were used, as they are less sensitive to outliers and may provide a more accurate measure of central tendency in positively skewed distributions.^9^ The median RTs were computed for each participant, and then a Bayesian paired-samples *t* test was conducted for the alternative hypothesis that the population mean of the difference in median RTs is smaller than zero (or greater than zero for H2d and H3d). The difference in median RTs for each participant between congruent and incongruent trials (medianRT_inc_ − medianRT_con_) was then calculated for further testing of RT priming effects. The sample means of these difference scores were then compared across conditions (e.g., ∆RT_unhealthy_ > ∆RT_healthy_ in H2d) and are referred to as ∆RT. Similarly, ∆ER was defined as the priming effect for error rates, where ∆ER = ER_inc_ − ER_con_. For the calculation of error rates at the participant level, accuracy is recoded as 1 = incorrect and 0 = correct.

With regard to FCT analyses, p(unhealthy|most liked) refers to the conditional probability of choosing an unhealthy food in the unhealthy versus healthy food-choice trials when most liked food pairs were presented (see Fig. 3 for trial types). Accordingly, p(most liked|healthy) denotes the conditional probability of choosing a most liked food in the most liked versus least liked trials where healthy food pairs are presented, and p(most liked|unhealthy) indicates the conditional probability of choosing a most liked food on trials where the unhealthy food pairs were presented. Choices were recoded according to trial types to compute these probabilities. For example, in trials where participants chose between most liked and least liked foods, and the foods presented were healthy, choices were coded as 1 = most liked and 0 = least liked. Then, the mean was calculated and denoted the probability that participants chose a most liked food in these most liked versus least liked (healthy) choice trials, that is p(most liked|healthy). Probability values were calculated from the number of completed trials. The difference in priming effects (RTs only) between unhealthy and healthy most liked food prime trials is represented by ∆RT_unhealthy_ − ∆RT_healthy_.

### Preregistered analyses

Bayesian paired-samples *t* tests (Rouder, Speckman, Sun, Morey, & Iverson, 2009) employed a prior with the √2*/*2 scale parameter for the half-Cauchy distribution. Bayesian correlation pairs had a stretched beta prior with width γ *=* 1, which corresponds to a uniform prior (Wagenmakers, Verhagen, & Ly, 2016). Analyses were conducted separately for the online and laboratory data sets, and results were reported independently (see Study Setting section). The evidential value and hence interpretation of the results was exclusively based on Bayes factors, but frequentist statistics have also been reported (*a* = 0.05). H1, H2 and H3 were exclusively tested using directional Bayesian paired-samples *t* tests, as outlined below.

H1a. RT_con_ < RT_inc_ for non-food prime trials

H1b. ER_con_ < ER_inc_ for non-food prime trials

H2a. RT_con_ < RT_inc_ for food prime trials

H2b. RT_con_ < RT_inc_ for healthy food prime trials

H2c. RT_con_ < RT_inc_ for unhealthy food prime trials

H2d. ∆RT_unhealthy_ > ∆RT_healthy_ for most liked food primes

H3a. ER_con_ < ER_inc_ for food prime trials

H3b. ER_con_ < ER_inc_ for healthy food prime trials

H3c. ER_con_ < ER_inc_ for unhealthy food prime trials

H3d. ∆ER_unhealthy_ > ∆ER_healthy_ for most liked food primes

H4 was only examined via directional Bayesian correlation pairs, as shown below. The reported correlation coefficient was Pearson’s rho. Definitions of probabilities have been described in detail above (see Measures and Indices section).

H4a. ∆RT_healthy_ for most liked primes positively correlates with p(most liked|healthy)

H4b. ∆RT_unhealthy_ for most liked primes positively correlates with p(most liked|unhealthy)

H4c. ∆RT_unhealthy_ − ∆RT_healthy_ (for most liked primes) positively correlates with p(unhealthy|most liked)

As a contingent analysis plan, Bayes factors for H1, H2 and H3 in the opposite direction would also be reported if differences between means were descriptively in the unexpected direction, such as RT_con_ > RT_inc_ for food prime trials. The decision to report the positive one-sided tests would be based on descriptive values and not on Bayes factors, as support for the null in a directional Bayesian *t* test does not exclude the possibility that there is greater evidence for an effect in the opposite direction. For example, even if there is adequate evidence for H0 and the null hypothesis is preferred to the negative hypothesis (RT_con_ < RT_inc_), the positive hypothesis (RT_con_ > RT_inc_) may still be favoured over the null (Morey, 2014). More details about the preregistered analysis plan (e.g., software, data transformations, reported statistics, effect size calculation) can be found in the Supplementary Material.

### Data exclusions

Error rates in the APP were inspected for food and non-food prime trials separately, and participants with ERs greater or equal to 0.4 from within either set of trials were excluded from all respective analyses. This obviated the need for further inspection of the distribution of missed or inaccurate responses across conditions. The FCT data were inspected for missed responses, where participants did not respond within 1,500 ms. Analyses conducted for H4a, H4b, and H4c would not include participants who had more than 50% of missed trials across the two blocks in any trial type examined under H4 (i.e., <16 out of 32 trials).

Data were also inspected for timing delays in trial events in the APP due to the possible occurrences of technical issues during online testing (e.g., slow broadband). Timing delays were defined as trial events that last two or more screen refreshes than originally programmed. The trial events that were inspected were the presentation of the prime (233 ms) and mask (17 ms), and trials with timing delays would be removed from analyses. If a participant had more than 25% of trials removed, they would then be excluded from all analyses.

## Results

### Sample characteristics

**Fig. 4:**
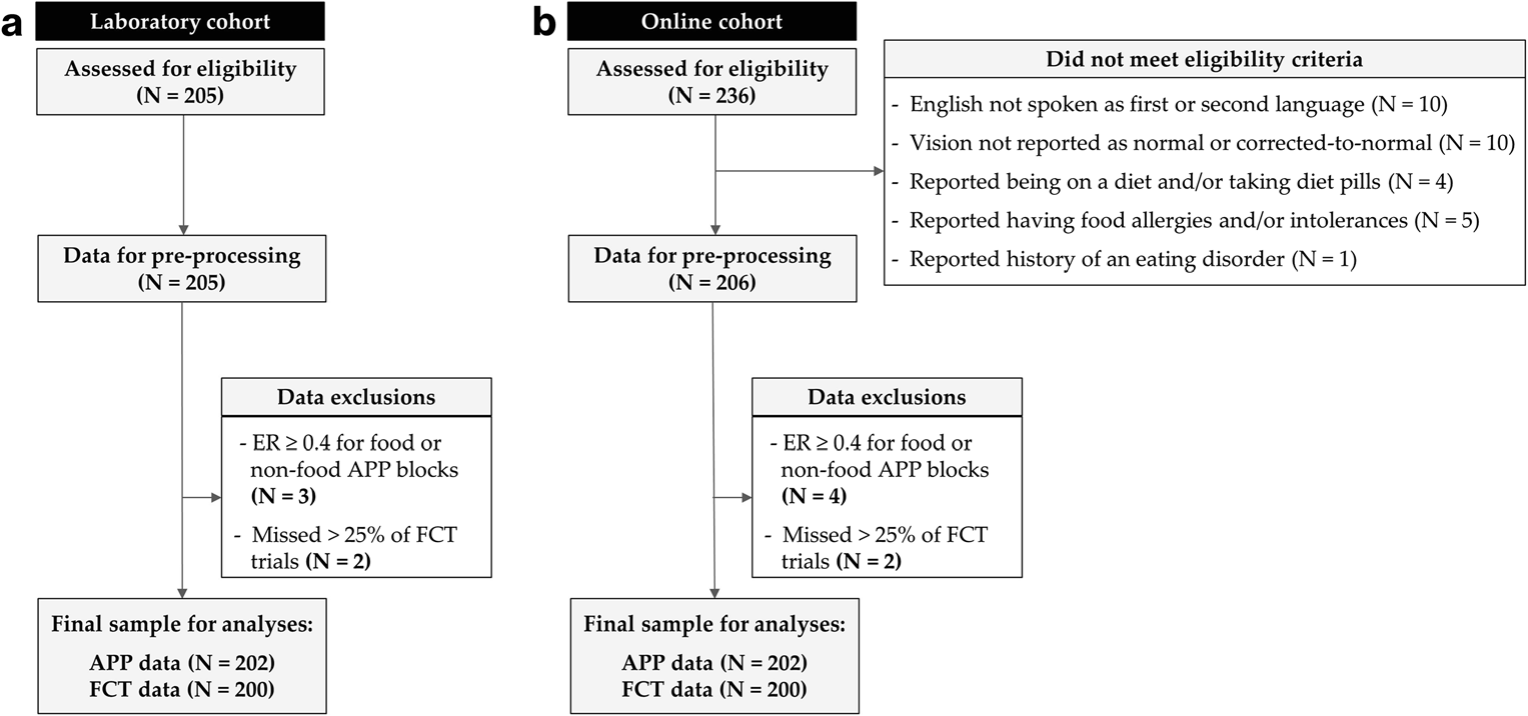
Flow diagram of recruitment and participant-level data exclusions. **a** A total of 205 participants were recruited from Cardiff University and completed the study in the laboratory. Three participants were excluded because their mean error rate in the food or non-food prime APP blocks was greater or equal to 0.40. Two participants were excluded from FCT analyses because they had more than 25% of missed trials overall. **b** Excluding dropouts, a total of 236 participants were recruited via Prolific and were assessed for eligibility, while 30 participants did not meet the eligibility criteria. The data from 206 participants were first inspected for timing delays in the APP, and the proportion of delayed trials was very low (1–5) only for nine participants and were discarded. Four participants were excluded due to error rates in the APP (≥0.4), and two participants were excluded because they had missed more than 25% of FCT trials. All exclusions were in accordance with preregistered criteria. *Note.* ER = error rate; APP = affective priming paradigm; FCT = food-choice task

The final sample size for APP analyses (H1–H3) was 202 for both the laboratory and online cohorts (see Fig. 4). Due to *n*_max_ not being reached for FCT analyses (H4) after data exclusions, additional participants were recruited, resulting in different sample sizes for APP and FCT analyses. The final sample size for FCT analyses (H4) was 200 for both cohorts. Using preregistered data quality assurance measures (see Follow-Up Study Questionnaire section), we found that, descriptively, the online study setting overall matched the controlled laboratory environment in terms of data quality (see Data Quality Checks in the Supplementary Material).

Descriptive statistics of demographics and other sample characteristics are shown in Table 1. Overall, the laboratory and online cohorts were approximately matched, but online testing generated a more diverse sample in terms of gender and age. Participants in both cohorts were generally not very hungry at the time of the study. In the laboratory sample, 58% of participants self-reported eating 1–3 hours before the study, and 21% of participants had a meal just before the study (“Less than 1 hour ago”). In the online sample, 53.5% of participants self-reported eating 1–3 hours before the study, and 21.2% of participants had a meal less than 1 hour before the study. A total of 161 participants from the laboratory cohort (80.5%) reported that they were not following any specific diet, while only 20 (10%) were vegetarian and 12 pescatarian (6%). Similarly, the online cohort consisted of 177 participants who did not follow a specific diet (89.4%), and only 14 participants reported a vegetarian diet (7.1%). A noteworthy difference between the two cohorts was participants’ BMI, as calculated by self-reported height and weight. The mean BMI in the online cohort trends towards the overweight category (≥25 kg/m^2^). The average FCQ-T-r total scores indicate that neither the laboratory, or online cohorts had “clinically relevant” levels of trait food cravings (Meule, 2018).

**Table 1.** Descriptive statistics of sample characteristics for laboratory and online cohorts

	Laboratory cohort		Online cohort	
	*Mean*	*SD*	*Mean*	*SD*
Age (years)	20.85	4.50	33.53	11.96
Gender (% female)	82.00	38.52	56.57	49.96
Ethnicity (% White)	84.50	36.28	84.85	35.95
Hunger (1–9)	5.03	2.34	4.55	2.50
FCQ-T-r total score	44.20	10.00	42.97	11.93
BMI (kg/m^2^)	22.59	3.48	25.17	5.88
Liking for non-food stimuli	93.30	10.37	89.23	15.70
Liking for “most liked” unhealthy foods	86.36	16.78	82.81	24.08
Liking for “least liked” unhealthy foods	−61.81	35.15	−55.87	38.55
Liking for “most liked” healthy foods	87.94	14.07	84.86	18.16
Liking for “least liked” healthy foods	−65.34	29.50	−62.45	32.36
Perceived healthiness of “healthy” foods	7.38	0.69	7.33	0.86
Perceived healthiness of “unhealthy” foods	2.26	0.74	2.44	0.89

*Note*. The descriptive statistics shown in this table excluded two participants from the online cohort who did not complete the last parts of the study (i.e., questionnaires). Participants from both cohorts who were excluded from confirmatory analyses were also not included. The sample sizes for the variables presented here are 200 and 198 for laboratory and online cohorts, respectively. Ethnicity was not provided (“do not wish to answer”) by two participants in the online cohort. Calculated BMI was considered invalid for four participants in the laboratory cohort and three participants in the online cohort (<15 or >60). Hunger was measured on a 9-point Likert scale (1 = *not at all* to 9 = *very*). Liking was measured using a visual analogue scale that ranged from −100 (*strongly dislike*) to 100 (*strongly like*), and perceived healthiness ranged from 1 (*very unhealthy*) to 9 (*very healthy*). FCQ-T-r = Food Cravings Questionnaire–Trait-reduced

### Findings from preregistered analyses

#### Interpretation of outcomes

The relative evidence for the confirmatory hypotheses was interpreted based on Bayes factors. BF_10_ denotes evidence for the alternative hypothesis (H1) compared with the null (H0), and BF_01_ reflects the relative evidence for the null. We have adopted the guidelines reported in Lee and Wagenmakers (2013) to describe the *strength* of relative evidence for each hypothesis. Regarding calculated effect sizes (Cohen’s *d*_av_), we acknowledge that commonly employed benchmarks are often arbitrary and require careful consideration of the specific research context and relevant literature (Lakens, 2013). Here we follow previous guidelines on Cohen’s *d* (Cohen, 1988) for ease of interpretation.

#### Manipulation check

**Fig. 5:**
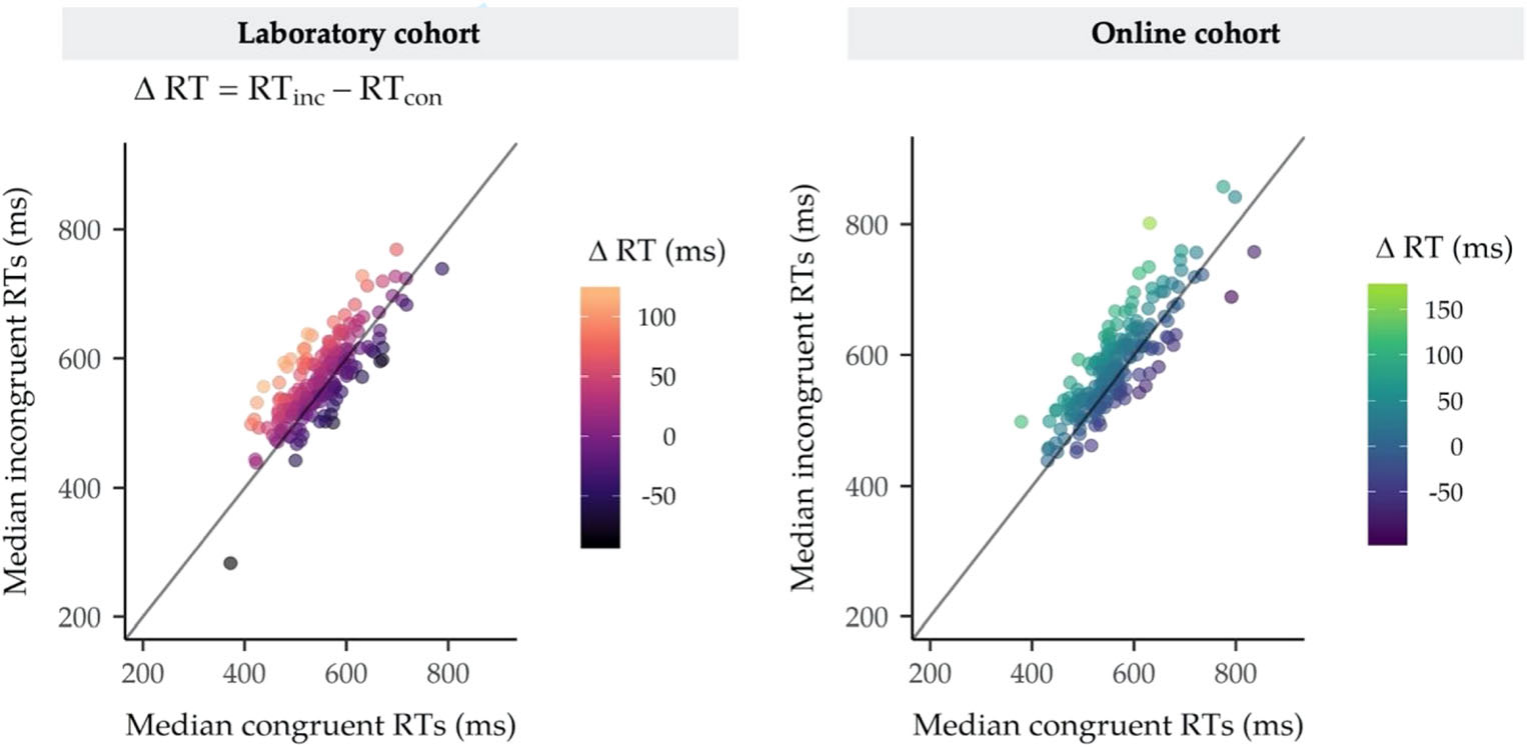
Plots showing the observed reaction time priming effects for non-food primes in the laboratory and online cohorts. The scatterplots show the individual median reaction times (RTs) calculated for non-food prime trials at the participant level and how they differ for each level of affective congruence. As expected, participants were faster to respond on congruent compared with incongruent trials (H1a) in both the laboratory and online cohorts. The RT priming effect (∆RT) was calculated as the difference between incongruent and congruent RTs (RT_inc_ − RT_con_). Any value shown above the unity line on these plots corresponds to a positive priming effect, whereby incongruent RTs (*y*-axis) are larger compared with congruent RTs (*x*-axis). The magnitude of ∆RT (ms) can be visualized using the colour gradient applied to plotted data points

We first report the results of the manipulation check for the APP as stated in H1 (i.e., non-food priming effects). *Extreme* evidence was obtained for the expected RT priming effects on non-food prime trials (H1a), as presented in Tables 2 and 3. In the laboratory cohort, participants were faster to respond in congruent (*M* = 549.5ms, *SD* = 64.8 ms) compared with incongruent non-food trials (*M* = 568.5 ms, *SD* = 63.2 ms) [*d*_av_ = −0.30, 95% CI for *d*_*av*_ *= −*0.39, −0.21]. This relatively small effect was replicated in the online cohort, with participants having, on average, lower median RTs on congruent (*M* = 563.6 ms, *SD* = 71.4 ms) rather than incongruent non-food trials (*M* = 585.9 ms, *SD* = 73.3 ms) [*d*_av_ = −0.31, 95% CI for *d*_av_ *= −*0.39, −0.23]. RT priming effects from non-food prime trials are shown in Fig. 5.

**Table 2:** Statistical test results for hypotheses H1 to H3 from the laboratory cohort (*N* = 202)

Hypothesis	Log(BF_10_)	*t*(201)	*p*	Evidence interpretation
H1a. RT_con_ < RT_inc_(non-food primes)	18.77	−6.81	<.001	*Extreme e*vidence for H1
H1b. ER_con_ < ER_inc_(non-food primes)	7.97	−4.57	<.001	*Extreme* evidence for H1
H2a. RT_con_ < RT_inc_(all foods)	37.11	−9.83	<.001	*Extreme* evidence for H1
H2b. RT_con_ < RT_inc_(healthy foods)	24.06	−7.74	<.001	*Extreme* evidence for H1
H2c. RT_con_ < RT_inc_(unhealthy foods)	28.24	−8.44	<.001	*Extreme* evidence for H1
H2d. ∆RT_unhealthy_ > ∆RT_healthy_ (most liked)	−1.53	1.00	.159	*Moderate* evidence for H0
H3a. ER_con_ < ER_inc_(all foods)	29.19	−8.59	<.001	*Extreme* evidence for H1
H3b. ER_con_ < ER_inc_(healthy foods)	19.50	−6.95	<.001	*Extreme* evidence for H1
H3c. ER_con_ < ER_inc_(unhealthy foods)	22.66	−7.50	<.001	*Extreme* evidence for H1
H3d. ∆ER_unhealthy_ > ∆ER_healthy_ (most liked)	−1.35	1.14	.128	*Moderate* evidence for H0

*Note*. Evidence is interpreted for the alternative hypothesis (H1) compared with the null (H0) and vice versa. The effect size is given by Cohen’s *d*_av_. Log(BF_10_) = natural logarithm of BF_10_; for example, BF_10_ > 10 is equivalent to Log(BF_10_) > 2.3, and BF_10_ < 1/10 is equivalent to Log(BF_10_) < −2.3; BF_10_ > 100 is represented by Log(BF_10_) > 4.61. Statistical tests for all hypotheses and related abbreviations are explained in the Analyses section

**Table 3:** Statistical test results for hypotheses H1 to H3 from the online cohort (*N* = 202)

Hypothesis	Log(BF_10_)	*t*(201)	*p*	Evidence interpretation
H1a. RT_con_ < RT_inc_(non-food primes)	24.94	−7.89	<.001	*Extreme* evidence for H1
H1b. ER_con_ < ER_inc_(non-food primes)	4.07	−3.52	<.001	*Very strong* evidence for H1
H2a. RT_con_ < RT_inc_(all foods)	25.92	−8.05	<.001	*Extreme* evidence for H1
H2b. RT_con_ < RT_inc_(healthy foods)	14.77	−6.05	<.001	*Extreme* evidence for H1
H2c. RT_con_ < RT_inc_(unhealthy foods)	17.70	−6.62	<.001	*Extreme* evidence for H1
H2d. ∆RT_unhealthy_ > ∆RT_healthy_ (most liked)	−1.98	0.62	.269	*Moderate* evidence for H0
H3a. ER_con_ < ER_inc_(all foods)	6.87	−4.30	<.001	*Extreme* evidence for H1
H3b. ER_con_ < ER_inc_(healthy foods)	7.93	−4.56	<.001	*Extreme* evidence for H1
H3c. ER_con_ < ER_inc_(unhealthy foods)	3.39	−3.30	<.001	*Strong* evidence for H1
H3d. ∆ER_unhealthy_ > ∆ER_healthy_ (most liked)	−2.65	−0.13	.553	*Strong* evidence for H0

*Note*. Evidence is interpreted for the alternative hypothesis (H1) compared with the null (H0) and vice versa. The effect size is given by Cohen’s *d*_av_. Log(BF_10_) = natural logarithm of BF_10_; for example, BF_10_ > 10 is equivalent to Log(BF_10_) > 2.3, and BF_10_ < 1/10 is equivalent to Log(BF_10_) < −2.3; BF_10_ > 100 is represented by Log(BF_10_) > 4.61. Statistical tests for all hypotheses and related abbreviations are explained in the Analyses section

In support of H1b, a small priming effect for error rates in the expected direction was observed in both samples. In the laboratory cohort, there was *extreme* evidence for lower error rates (proportion of errors) on congruent (*M* = 0.06, *SD* = 0.07) compared with incongruent non-food trials (*M* = 0.09, *SD* = 0.08) [*d*_av_ = −0.35, 95% CI for *d*_av_ *=* −0.51, −0.20; *W* = 3,477.50, *p*_*W*_ < .001].^10^ Similarly, in the online cohort there was *very strong* evidence for error rates being reduced from congruent (*M* = 0.04, *SD* = 0.05) to incongruent non-food trials (*M* = 0.06, *SD* = 0.09) [*d*_av_ = −0.32, 95% CI for *d*_av_ *=* −0.50, −0.14; *W* = 3,063.00, *p*_*W*_ < .001]. These findings confirm the success of the manipulation check.

#### Food priming effects

##### Findings from primary laboratory cohort

**Fig. 6:**
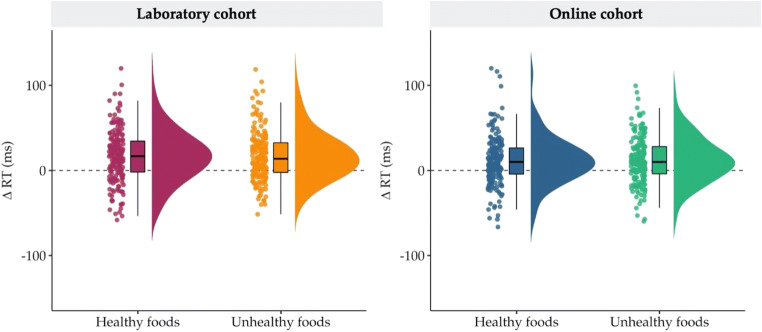
Plots showing the observed reaction time priming effects for healthy and unhealthy food primes in the laboratory and online cohorts. The raincloud plots show the individual median reaction times (RTs) from trials where healthy and unhealthy foods were presented. ∆RT (ms) refers to the difference between congruent and incongruent trials, where positive scores reflect an RT priming effect. The line drawn at *y* = 0 shows that for both laboratory and online cohorts the distributions of participants’ median RTs were overall positive. The spread of the data indicates that, on average, priming effects for both healthy and unhealthy foods were reliably observed across participants

The results of all statistical tests for preregistered hypotheses H2 and H3 from the laboratory cohort (*N* = 202) are presented in Table 2. As preregistered, RTs (and RT difference scores) for all comparisons under H2 were log-transformed due to the violation of the normality assumption for H2a, H2c, and H2d. Bayesian and frequentist *t* tests were therefore conducted using log-transformed data. We report the nontransformed sample means of median RTs here for a more convenient interpretation of mean differences. We obtained *extreme* evidence for a small RT priming effect across food prime trials (H2a), as RTs were on average faster on congruent (*M* = 549.1 ms, *SD* = 58.5 ms) compared with incongruent trials (*M* = 565.4 ms, *SD* = 60.2 ms) [*d*_av_ = −0.27, 95% CI for *d*_av_ *=* −0.33, −0.21]. A small effect was observed in healthy food prime trials (H2b), whereby RTs were faster on congruent (*M* = 549.2 ms, *SD* = 59.6 ms) compared with incongruent trials (*M* = 565.6 ms, *SD* = 61.5 ms) [*d*_av_ = −0.27, 95% CI for *d*_av_ *=* −0.34, −0.19]. There was *extreme* evidence for a small RT priming effect across unhealthy food prime trials (H2c), as shown by RTs in congruent (*M* = 549.3 ms, *SD* = 59.3 ms) and incongruent trials (*M* = 566.3 ms, *SD* = 61.1 ms) [*d*_av_ = −0.28, 95% CI for *d*_av_ *=* −0.35, −0.21]. Observed priming effects for healthy and unhealthy food prime trials have been visualized using raincloud plots (Allen, Poggiali, Whitaker, Marshall, & Kievit, 2018, 2019; see Fig. 6). In contrast to our prediction that the RT priming effect would be greater for unhealthy compared with healthy most liked food prime trials (H2d), there was *moderate* evidence for the null hypothesis compared with the alternative. The RT priming effect for unhealthy most liked food primes (∆RT_unhealthy_; *M* = 17.8 ms, *SD* = 43.8 ms) was *not* greater than the RT priming effect for healthy most liked food primes (∆RT_healthy_; *M* = 13.6 ms, *SD* = 48.1 ms) [*d*_av_ = 0.08, 95% CI for *d*_av_ *=* −0.07, 0.22].

As explained in the Hypotheses section, we expected support for any observed priming effects to be evident in both speed-related and accuracy-related hypotheses. There was *extreme* evidence for a medium ER priming effect across food prime trials (H3a). Participants made fewer errors on congruent (*M* = 0.06, *SD* = 0.04) compared with incongruent food prime trials (*M* = 0.09, *SD* = 0.07) [*d*_av_ = −0.54, 95% CI for *d*_av_ *=* −0.68, −0.41; *W* = 2,581.50, *p*_*W*_ < .001]. A medium effect was also observed in healthy food prime trials (H3b), as error rates were lower on congruent (*M* = 0.06, *SD* = 0.05) relative to incongruent trials (*M* = 0.09, *SD* = 0.07) [*d*_av_ = −0.49, 95% CI for *d*_av_ *=* −0.63, −0.34; *W* = 3,960.00, *p*_*W*_ < .001]. There was also *extreme* evidence for a medium ER priming effect in unhealthy food prime trials (H3c). We found the expected differences in error rates between congruent (*M* = 0.06, *SD* = 0.05) and incongruent unhealthy food prime trials (*M* = 0.09, *SD* = 0.07) [*d*_av_ = −0.51, 95% CI for *d*_av_ *=* −0.66, −0.37; *W* = 2,849.00, *p*_*W*_ < .001]. Priming effects for error rates were in the expected direction across food prime trials, and therefore we can conclude that any RT effects were not observed due to strategic responding or speed–accuracy trade-offs. Contrary to predictions about differences in ER priming effects between healthy and unhealthy most liked food primes (H3d), there was *moderate* evidence for the null compared with the alternative hypothesis. The ER priming effect was *not,* on average, greater for unhealthy (*M* = 0.02, *SD* = 0.07) compared with healthy most liked food primes (*M* = 0.01, *SD* = 0.08) [*d*_av_ = 0.10, 95% CI for *d*_av_ *= −*0.07, 0.27; *W* = 8822.00, *p*_*W*_ = .105].

##### Direct replication: Findings from online cohort

The results of all statistical tests for preregistered hypotheses H2 and H3 from the online cohort (*N* = 202) are presented in Table 3. RTs for all comparisons under H2 were log-transformed (logRTs) due to the violation of the normality assumption for H2b, in line with the preregistered analysis plan, and nontransformed sample means are reported here for convenience. Reaction time and error rate priming effects were replicated in the online cohort. First, there was *extreme* evidence for a small RT priming effect across food prime trials (H2a), as on average RTs on congruent trials (*M* = 568.6 ms, *SD* = 71.3 ms) were faster compared with RTs on incongruent food prime trials (*M* = 580.5 ms, *SD* = 70.7 ms) [*d*_av_ = *−*0.18, 95% CI for *d*_av_ *= −*0.22, *−*0.13]. A small RT priming effect was also observed in healthy food prime trials (H2b). RTs were faster on congruent (*M* = 568.4 ms, *SD* = 71.4 ms) compared with incongruent healthy food prime trials (*M* = 580.1 ms, *SD* = 74.0 ms) [*d*_av_ = *−*0.17, 95% CI for *d*_av_ *= −*0.22, *−*0.11]. *Extreme* evidence was obtained for a small RT priming effect in the expected direction for RTs on congruent (*M* = 568.7 ms, *SD* = 73.6 ms) and incongruent (*M* = 581.0 ms, *SD* = 71.0 ms) unhealthy food prime trials [H2c; *d*_av_ = *−*0.18, 95% CI for *d*_av_ *= −*0.24, −0.13]. The results from laboratory and online cohorts converge for H2d as well, as there was *moderate* evidence that the RT priming effect for most liked unhealthy foods (∆RT_unhealthy_; *M* = 14.5 ms, *SD* = 44.7 ms) was *not* greater than the RT priming effect for most liked unhealthy foods (∆RT_healthy_; *M* = 12.8 ms, *SD* = 43.0 ms) [*d*_av_ = 0.05, 95% CI for *d*_av_ *= −*0.10, 0.19].

In line with the findings from the laboratory cohort, priming effects were observed in terms of error rates ruling out the possibility of strategic performance trade-offs. However, in the online cohort, we found small, and not medium, ER food priming effects. There was *extreme* evidence for a small ER priming effect across food prime trials (H3a). Error rates on congruent trials (*M* = 0.05, *SD* = 0.05) were, on average, lower compared with error rates on incongruent food prime trials (*M* = 0.06, *SD* = 0.07) [*d*_av_ = −0.28, 95% CI for *d*_av_ *=* −0.40, −0.15; *W* = 4,238.00, *p*_*W*_ < .001]. In healthy food prime trials (H3b), results for error rates on congruent (*M* = 0.05, *SD* = 0.05) and incongruent trials (*M* = 0.06, *SD* = 0.07) were in the same direction [*d*_av_ = -0.30, 95% CI for *d*_av_ *=* −0.43, −0.17; *W* = 3,110.00, *p*_*W*_ < .001]. *Strong* evidence was also obtained for a small ER priming effect in unhealthy food prime trials (H3c), as error rates were on average lower on congruent (*M* = 0.05, *SD* = 0.05) compared with incongruent trials (*M* = 0.06, *SD* = 0.08) [*d*_av_ = −0.23, 95% CI for *d*_av_ *=* −0.37, −0.09; *W* = 4,942.50, *p*_*W*_ < .001]. Consistent with the results for H3d in the laboratory cohort, there was *strong* evidence that the ER priming effect was *not* greater for unhealthy (*M* = 0.01, *SD* = 0.08) compared with healthy most liked food primes (*M* = 0.01, *SD* = 0.07) [*d*_av_ = −0.01, 95% CI for *d*_av_ *=* −0.15, 0.13; *W* = 6,302.50, *p*_*W*_ = .779].

#### Food-choice behaviour

Bayesian correlation pairs for hypotheses H4a and H4b have yielded conclusive evidence regarding the absence or presence of the expected linear positive correlations. There was *strong* evidence that the probability of choosing a most liked food over a least like food from within a pair of healthy food stimuli (*M* = 0.97, *SD* = 0.06) did *not* positively correlate with the RT priming effect in healthy food prime trials [H4a; BF_01_ = 21.89; *r* = −.073, *p* = .849, 95% CI = −0.210, 0.066]. Similarly, there was *strong* evidence that the probability of choosing a most liked over a least liked food from within a pair of unhealthy food stimuli (*M* = 0.96, *SD* = 0.05) did *not* positively correlate with the RT priming effect in unhealthy food prime trials [H4b; BF_01_ = 15.98; *r* = −.035, *p* = .686, 95% CI = −0.172, 0.105]. *Moderate* evidence was obtained for the null compared with the alternative hypothesis for H4c. The probability of choosing an unhealthy over a healthy most liked food (*M* = 0.60, *SD* = 0.28) did *not* positively correlate with the difference in RT priming effects between unhealthy and healthy most liked food prime trials [H4c; BF_01_ = 6.70; *r* = .041, *p* = .283, 95% CI = −0.098, 0.179].

In the online cohort, there was *moderate* evidence for the lack of a positive correlation between the probability of choosing a most liked food over a least like food from within a pair of healthy food stimuli, or p(most liked|healthy) (*M* = 0.96, *SD* = 0.08), and the RT priming effect in healthy most liked food prime trials [H4a; BF_01_ = 4.88; *r* = .061, *p* = .195, 95% CI = −0.078, 0.198]. There was also *moderate* evidence for the absence of a positive linear correlation between the probability of choosing a most liked over a least liked food from within a pair of unhealthy food stimuli, or p(most liked|unhealthy) (*M* = 0.94, *SD* = 0.10), and the RT priming effect in unhealthy food prime trials [H4b; BF_01_ = 7.62; *r* = .032, *p* = .328, 95% CI = −0.108, 0.170]. As a further validation of the FCT, both probabilities of choosing a most liked food item in the most liked vs least liked trials were very high and above 0.5. Consistent with the laboratory cohort, there was *moderate* evidence for the lack of a positive linear correlation between the probability of choosing an unhealthy over a healthy most liked food, or p(unhealthy|most liked) (*M* = 0.55, *SD* = 0.33), and the difference in RT priming effects between unhealthy and healthy most liked food prime trials [H4c; BF_01_ = 6.43; *r* = .044, *p* = .270, 95% CI = −0.096, 0.181].

## Discussion

The primary aim of this study was to assess the utility of the affective priming paradigm (APP) as an indirect measure of food liking and related choice behaviour. Using a variant of the APP that requires evaluative categorization that is semantically unrelated to the content of the primes, participants responded as quickly and as accurately as possible to the valence of word targets. Affective congruence was manipulated so that both healthy and unhealthy foods that had been selected as most liked or least liked via an initial rating task were paired with both positive and negative targets. After the APP, participants completed a binary food-choice task (FCT), and impulsive food-choice probabilities for different food pairs were measured. The three main research questions of the study were tested via preregistered confirmatory hypotheses in laboratory settings and replicated in a second cohort of participants who completed the experiment online. The findings for each of these research questions and their implications are discussed at length below, together with directions for future research.

### Can priming effects for foods be obtained with the APP?

Yes. In line with previous findings (e.g., Lamote et al., 2004), robust priming effects were observed across food prime trials (most liked and least liked foods) for both speed (RTs) and accuracy (ERs). Effects were also shown to be robust for both healthy and unhealthy foods (see Fig. 6), providing conclusive evidence that the APP can be used as an indirect measure of food liking. The reaction time (RT) priming effects were relatively small, and although effect sizes for direct comparisons are not commonly reported in the literature, mean differences between RTs on congruent and incongruent trials seem to be consistent with previous studies that employed similar paradigms (e.g., Lamote et al., 2004; Roefs, Herman, et al., 2005a; Verhulst et al., 2006). The interpretation of the findings was strengthened by the success of the manipulation check for the APP, which assessed priming effects for most liked non-food stimuli. Importantly, all results from the laboratory cohort (*N* = 202) were directly replicated in the online cohort (*N* = 202). Statistical tests were repeated under different data aggregation/reduction criteria adopted from previous literature (Lamote et al., 2004; Verhulst et al., 2006) in order to establish the robustness of the observed priming effects. There were no discrepancies between the results based on the preregistered analysis plan and the alternative analyses (see Table S4 in the Supplementary Material), which suggests that our findings were not influenced by the aggregation and outlier removal criteria employed in this study.

### Is the APP sensitive to cognitive components of food attitudes?

Possibly not, but if cognitive components have an effect on task performance, this is likely to be small. The sensitivity of the APP in capturing both affective and cognitive components of attitudes was investigated by comparing the RT and ER priming effects for healthy and unhealthy most liked foods. Previous literature employing the APP indicates that a greater priming effect for healthy (or low-fat) compared with unhealthy (or high-fat) foods may indicate that the APP taps into cognitive components of attitudes (i.e., food healthiness). For example, Roefs, Stapert, et al. (2005b) suggested that health concerns may have determined observed priming effects in two groups of participants who differed in terms of BMI and dietary restraint (Experiment 2). Specifically, the authors report that participants in both groups showed a “preference” for low-fat over high-fat foods.^11^ Previous research also suggests that priming effects are sensitive to changes in context/environment and attentional focus, such as participants performing the study in a local hospital instead of a laboratory and experimentally manipulating the focus of participants’ attention on either the palatability or healthiness of food stimuli before the task (Roefs et al., 2006; Roefs, Stapert, et al., 2005b).

Although we obtained conclusive evidence that healthiness did not influence the magnitude of the RT and ER priming effects for most liked foods, we could not conclude that this is because the APP is only sensitive to affective components of attitudes. Future research could assess how healthiness affects the relative strength of observed priming effects when individuals self-report a preference of healthy over unhealthy foods, which could be attributed to social desirability or health concerns. In both study cohorts there were no descriptive differences in explicit liking between healthy and unhealthy most liked foods that could transfer to the APP. Although healthiness may not affect priming effects in an explicit manner that would undermine its validity as an indirect measure of liking—as, for example, when these reflect concerns related to health, weight-related goals or social norms (Czyzewska & Graham, 2008)—healthiness could influence task outcomes through implicit healthiness attributes that are automatically retrieved from memory (e.g., see Rangel, 2013; Trendel & Werle, 2015). We specifically assumed that if individuals had greater automatic affective reactions towards unhealthy foods (e.g., “unhealthy = tasty” intuition; Raghunathan, Naylor, & Hoyer, 2006; also see Werle, Trendel, & Ardito, 2013), this would manifest as a positive difference in the magnitude of RT/ER priming effects, even when food primes were matched on explicit liking.^12^ However, even if unhealthy food primes induce stronger affective reactions compared with healthy food primes, there is not enough evidence to suggest that the APP would capture such subtle differences. The seminal study by Lamote et al. (2004) actually indicated that prime extremity (e.g., moderate vs. strong liking) does not influence observed priming effects, which could mean that only the overall valence of the primes (liked/positive, disliked/negative) determines task outcomes.

Overall, our findings are consistent with the study by Becker et al. (2015), which did not report any differences for the food prime contrasts (healthy, unhealthy, control) in their affective priming paradigm (Study 2). Similarly, Roefs, Herman, et al. (2005a) provided evidence for a priming effect for palatable (most liked) and unpalatable (least liked) foods, but found that fat content did not influence the results. Our results suggest that affective priming effects for most liked food primes in this study were not influenced by healthiness in any observable manner (i.e., differences between ΔRTs or ΔERs in H2d and H3d). Nevertheless, we recommend that the sensitivity of the APP to affective and cognitive components of attitudes is explored further in target populations (e.g., restrained eaters and individuals with a BMI in the overweight and/or obese category; Cserjesi, De Vos, & Deroost, 2016; Papies, Stroebe, & Aarts, 2009; Roefs, Herman, et al., 2005a; Roefs, Stapert, et al., 2005b).

### Are priming effects associated with impulsive food-choice behaviour?

No, at least in this design. A novel contribution of this study was the investigation of impulsive food choices using a binary reaction time task, adapted from previous literature (Veling et al., 2017; Zoltak et al., 2018; but also see Verhulst et al., 2006). Food liking has a paramount role in dietary choices, which are often impulsive and not guided by deliberate thoughts (Eertmans et al., 2001; Veling et al., 2017). If positive affective reactions towards foods can influence impulsive food choices (Zoltak et al., 2018), priming effects obtained via the APP could in theory be associated with the probability of choosing appetitive foods under different conditions, such as choosing a most liked unhealthy food when most liked and least liked foods are presented. Alternatively, if we assume that impulsive choices are driven by “wanting” and not “liking” for food cues in the environment (Berridge et al., 2010), performance in the APP would not be predictive of choice behaviour in the laboratory.

Confirmatory analyses provided conclusive evidence for the *absence* of positive linear correlations between RT priming effects for most liked foods and the probability of choosing a most liked food from both healthy and unhealthy items. These null findings were replicated in the online cohort. A potential limitation of the FCT design for *most liked versus least liked* food pairs was that food-choice probabilities were very high and there was not enough variability in participants’ responses, and that could have influenced the tested correlations. An even more meaningful question was whether the difference between RT priming effects for most liked healthy and unhealthy foods was associated with the probability of choosing an unhealthy food in trials where most liked healthy and unhealthy foods were presented. Again, however, there was strong support for the null hypothesis compared with the alternative in both laboratory and online cohorts. One notable methodological difference between the two cohorts was that in online testing settings the FCT did not involve offering participants food items for consumption, which meant that impulsive food choices were not consequential. However, as mentioned above, results converged fully between the two cohorts and although their choices were not consequential, participants in the online cohort had very high probabilities of selecting most liked foods on FCT trials where healthy and unhealthy food pairs were presented.

One issue that remains unclear is whether the strict time limit for these food choices between two most liked food items caused a choice uncertainty that could not be easily resolved, leading some participants to respond randomly or arbitrarily in these FCT trials. Follow-up experiments could measure both impulsive and deliberate food choices or use alternative time windows (e.g., short vs. long) and self-report questionnaires to discard alternative explanations for the absence of a positive correlation between RT priming effect difference scores and food-choice probabilities. We also recommend that future studies employ informative analysis priors for Bayesian correlations, as we believe that if a relatively *weak* relationship exists between priming effects and food-choice behaviour, the number of observations required to capture this would be very large with the current choice of prior distribution (e.g., see Fig. S1b in the Supplementary Material).

### Considerations for future research

Most participants in both laboratory and online cohorts were healthy-weight individuals with self-reported frequency and intensity of food craving experiences that did not indicate unhealthy eating behaviours, such as binge eating (Meule, 2018). It is possible that the absence of differences between RT priming effects for most liked healthy and unhealthy foods was because participants, on average, did not have stronger affective, or hedonic, reactions towards unhealthy foods. This research question could be addressed in a sample of individuals that are overweight and/or obese or show eating disorder symptomatology. In such cases, the distinction between cognitive and affective components of food attitudes may be more informative due to increased approach and/or attentional bias towards appetitive cues and the conflict of this bias with health-related goals, such as losing weight (Kakoschke et al., 2015). Accordingly, impulsive food choices that are driven by strong affective reactions towards unhealthy foods should be examined further in a representative sample of individuals that exhibit unhealthy eating behaviours, such as overeating.

Another next step in this line of research could be to employ different variants of the APP to disentangle theoretical explanations of priming effects and attempt to replicate and extend the presented findings. Affective priming effects can be explained by response competition/facilitation processes, as the primes can be defined as being congruent or incongruent to the required response to the target (Fazio & Olson, 2003; Wentura & Degner, 2010). Theoretically, however, it is also possible that the perception of the prime activates the “object-evaluation association” from memory, increasing the accessibility of valence for the targets when these are congruent with the prime compared with incongruent (see Fazio, 2001; Herring et al., 2013). In the evaluative categorization task, this distinction between the *encoding* and *response* perspectives cannot be inferred from observed priming effects. The pronunciation or naming task variant of the APP (see Herring et al., 2013, for discussion) involves responding to targets irrespective of their valence and can therefore exclude the *response* perspective from the explanation of any obtained priming effects.

The compatibility between the prime and the required response to the target may further reduce the “implicitness” of the measure if participants are aware of its effects. The standardized follow-up study questionnaire results (see Supplementary Material) showed that many participants were aware of the effects of affective congruence on their performance. For example, they believed that the content of the picture influenced their performance when the word they had to categorize was negative and the preceding picture depicted a food they liked the most. In addition to APP performance, potential confounds for primes and targets should be considered. Even in cases where robust priming effects fail to be obtained, participants’ individual explicit ratings of target valence and prime characteristics can have substantial diagnostic value and provide the basis for the investigation of individual differences. To bridge the gap between explicit and implicit measures of food attitudes, it may be worth pursuing the use of semistructured questionnaires that measure participants’ subjective awareness of performance differences in critical trials (e.g., congruent vs incongruent in the APP) as well as tailored stimulus selection (e.g., selecting primes via an initial rating task).

Exploratory analyses (see Data Quality Checks in Supplementary Material) also indicated that data from laboratory and online settings did not differ in terms of quality and precision. On the contrary, it is possible that participants in laboratory studies are more aware of experimental procedures, which could lead to increased bias in responses (e.g., demand characteristics; Podsakoff, MacKenzie, Lee, & Podsakoff, 2003). Where data quality assurance measures are in place (e.g., attention checks), online testing provides a fruitful avenue for studies requiring larger and more diverse samples and direct replications.

#### Open practices statement

All raw and processed data are publicly available at https://osf.io/73xfr. Study materials can be found at https://osf.io/sjcx7. All codes necessary for the reproduction of confirmatory analyses is publicly available at https://osf.io/73xfr. The Stage 1 protocol received in-principle acceptance on 20/02/2019 and was registered on the Open Science Framework at https://osf.io/y2tus.

## Electronic Supplementary Material

ESM 1 (PDF 708 kb)

## Footnotes

1 The robustness of the manipulation check has been demonstrated in a series of unregistered pilot experiments (see Paradigm Development at https://osf.io/z6nmx/).

2 Logical operators: ¬ = “not”; ⋁ = “or”; ⋀ = “and”

3 Prolific requires prescreening of participants, and the current country of residence was set to UK for two reasons: (1) consistency of subject pools between laboratory and online settings and (2) food brands included in the behavioural tasks might not have been popular outside the UK.

4 A £6.00/hr rate was used for both Prolific and EMS participants. On Prolific, the estimated time of completion was 45 minutes, and, in the laboratory, the study was expected to last 60 minutes due to the coordination of group testing settings and the time it would take to provide all participants with the chosen food items (see Procedure).

5 Questionnaire items may prime participants to pay attention to health-related or weight-related information, and therefore were presented after the behavioural tasks had been completed.

6 Because of the separate randomization of targets for food and non-food prime trials, certain targets may have appeared twice in a block.

7 We purposefully deviated from the previous literature on single-hand responses because of the possibility of learned associations (e.g., between the index/middle finger commands for “positive” and “negative” in the APP and food choices). Here, participants responded using both hands, by placing their index fingers on the “C” and “M” keys.

8 Random selection in steps of 100 ms.

9 Robustness checks were conducted to ensure that findings from confirmatory analyses were not sensitive to the choice of data exclusion and aggregation criteria (e.g., mean instead of median RTs). The preregistered robustness checks and the outcomes of all analyses can be found in the Supplementary Material.

10 In this section, *p*_*W*_ denotes the *p* value from the Wilcoxon signed-rank tests for clarity. In line with the preregistered analysis plan, these were conducted for tests under H3 if normality was violated (see Supplementary Material).

11 Note that the interaction between prime fat content and target affect (positive vs. negative) for palatable foods was not strong enough to contradict the present findings (η^2^ = 0.05, *p* = .09).

12 We could also presume that both healthiness and tastiness attributes form a learned evaluation that is automatically retrieved from memory. In this case, attitudes would be either positive or negative (healthiness and tastiness covary), without separate and accessible components.
