# Supplementary material for: The affective priming paradigm as an indirect measure of food attitudes and related choice behaviour
Source: Psychon Bull Rev. 2020 Jun 30;27(6):1397–415. doi: 10.3758/s13423-020-01764-1 (PMC7704519; doi:10.3758/s13423-020-01764-1)
Supplement: Supplementary file 1 — (PDF 708 kb) [file 13423_2020_1764_MOESM1_ESM.pdf]

Supplementary Material

Bayes factor design analysis

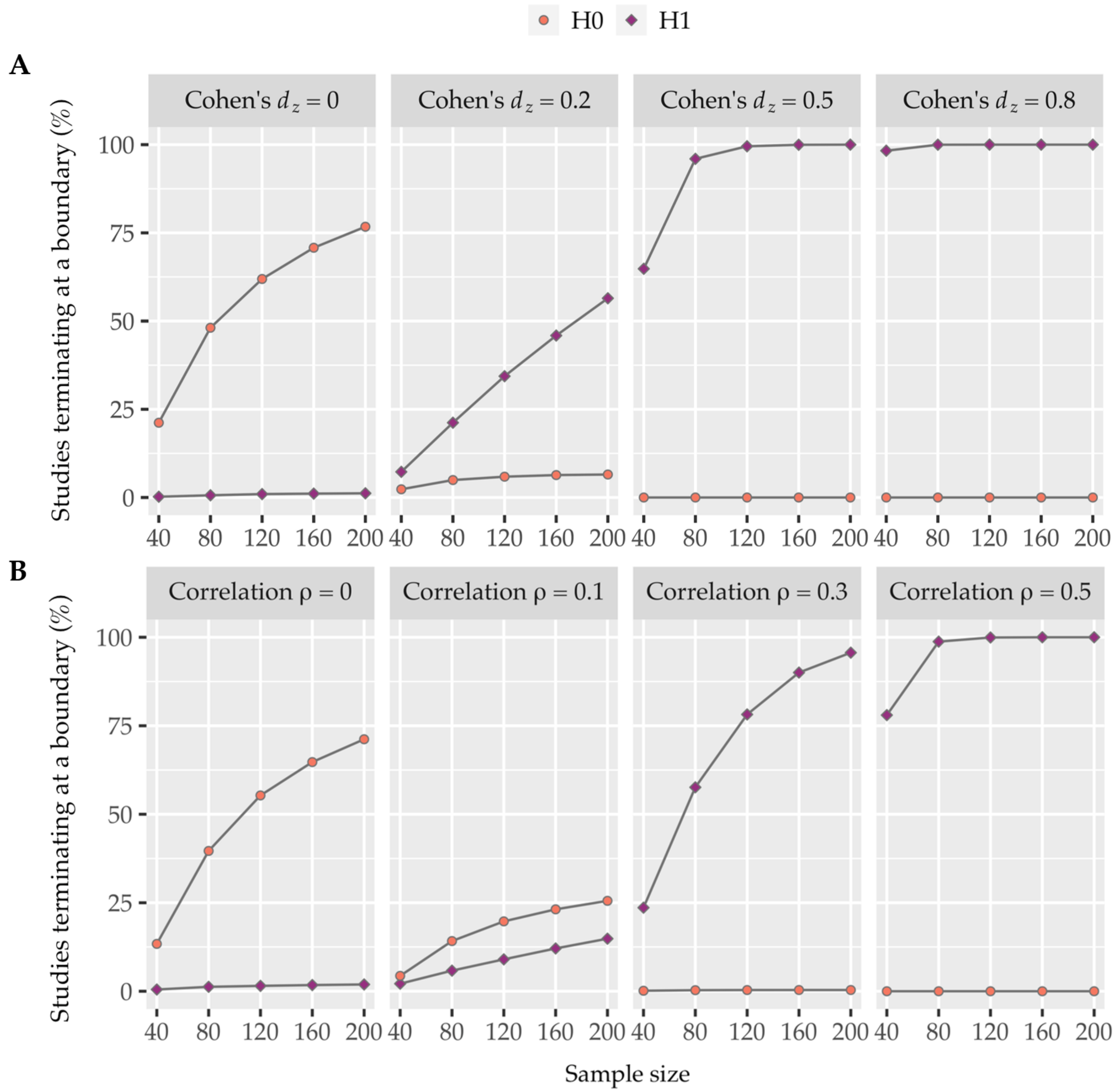

Figure S1: Bayes factor design analysis (BFDA) results for H0 and H1 at different sample and effect sizes in a simulated sequential design. The graphs show the percentage of simulated studies (10000) terminating at a boundary (H1 and H0) when the threshold is set to $BF_{01} \geq 10$ and $BF_{10} \geq 10$. BFDA has been conducted for the sample sizes of 40 ($nmin$), 80, 120, 160 and 200 ($nmax$). The Cohen's d_z and correlation ρ (rho) values reflect the potential of the true effect size being either zero (i.e., H0 is true), 'small' ($d_z = 0.2$; $\rho = 0.1$), 'medium' ($d_z = 0.5$; $\rho = 0.3$) or 'large' ($d_z = 0.8$; $\rho = 0.5$). These benchmarks are used for demonstration purposes only.

A. This panel shows the BFDA results for the planned directional Bayesian paired-samples t-tests (H1-H3). For H0, 77.04% of all simulated studies correctly terminate at the H0 boundary when *nmax* has been reached. However, the probability of obtaining false positive evidence is low, with only 1.5% of the studies incorrectly stopping at the H1 boundary. At n = 40, the probability is very low, with only 0.36% of studies incorrectly stopping at the H1 boundary. Assuming a small true effect size for H1 ($dz = 0.2$), at *nmax* 57.7% of simulated studies terminate at the correct H1 boundary and 5.9% of studies stop at the H0 boundary (i.e., probability of obtaining false negative evidence). Assuming a medium true effect size ($dz = 0.5$), at a sample size of 120, 99.7% of all studies correctly terminate at the H1 boundary and no studies (0%) stop at the H0 boundary. For a large true effect size ($dz = 0.8$), 80 participants would be adequate to correctly support H1 with 100% of simulated studies correctly reaching the H1 boundary. **B. This panel shows the BFDA results for the planned directional Bayesian correlations (H4).** Assuming the absence of a positive correlation (H0) at *nmax*, 71.19% of all simulated studies correctly terminate at H0 and 1.92% incorrectly stop at the H1 boundary. For a small true effect size ($\rho = 0.1$), only 14.85% of studies correctly terminate at H1 when *nmax* is reached and 25.54% of studies stop at H0. For a medium true effect size ($\rho = 0.3$), at *nmax* 95.65% of all studies correctly provide strong evidence for H1 and for a large effect size ($\rho = 0.5$) at the sample size 120, 99.94% of all simulated studies correctly terminate at the H1 boundary.

Targets in the affective priming paradigm

Word characteristics and ratings were obtained from the *EMOTE* database (Grühn, 2016). The sets of positive and negative words were matched as much as possible on emotionality, imagery, concreteness and familiarity, as shown below in Table S1. Negative targets are on average higher on arousal compared to positive targets and there is also a discrepancy for word frequency between the two sets of words.

Table S1: Word characteristics for positive and negative targets in the affective priming paradigm

	Positive targets		Negative targets	
	M	*SD*	*M*	*SD*
Frequency (BNC)	79.17	91.09	48.03	62.93
Valence	6.00	0.38	1.79	0.35
Arousal	2.28	0.57	4.65	1.58
Emotionality	4.63	0.98	5.00	0.86
Imagery	5.61	0.70	5.30	0.86
Concreteness	4.24	0.99	4.52	1.00
Familiarity	4.91	0.69	4.82	0.82

M: Mean; SD: Standard deviation; BNC: British National Corpus. *Note.* All word characteristics are scored on a scale from 1 to 7, apart from frequency (BNC).

Positive targets

1. HAPPY
2. SMILE
3. DREAM
4. BEAUTY
5. FRIEND
6. LOVE
7. PEACE
8. HEAVEN
9. PLEASURE
10. NICE
11. KIND
12. JOY
13. FREE
14. CHEER
15. HUMOUR
16. HUG
17. CUTE
18. RAINBOW
19. PARTY
20. SUNSET
21. ANGEL
22. KISS
23. LOYAL
24. LUCKY
25. LAUGHTER
26. BRAVE
27. INSPIRED
28. PROUD
29. WIN
30. TRAVEL
31. PRETTY
32. HOPE

Negative targets

1. AFRAID	9. JAIL	17. TRAUMA	25. GRIEF
2. HATE	10. HURT	18. STINK	26. UPSET
3. KILL	11. PAIN	19. BULLY	27. CRUEL
4. SICK	12. ANGRY	20. NASTY	28. AWFUL
5. EVIL	13. DEATH	21. RUDE	29. ABUSE
6. BAD	14. ALONE	22. CORPSE	30. DESPAIR
7. POISON	15. FEAR	23. INSULT	31. TERROR
8. MURDER	16. CORRUPT	24. SLAUGHTER	32. WAR

Food stimuli & food choice task setup

Descriptive & nutritional information of food stimuli

Stimuli were obtained from the food-pics online database (Blechert, 2019; Blechert, Meule, Busch, & Ohla, 2014) and Pixabay (https://pixabay.com/). Many food stimuli (i.e., branded foods) have been photographed and all stimuli were edited to have the same dimensions and a white background. The healthiness of the foods was considered based on what constitutes a healthy eating pattern, whereby the consumption of vegetables, fruits, grains and protein foods is encouraged, whereas added sugars, saturated fats and sodium intake should be limited (see https://perma.cc/M5CZ-44DZ). Nutritional information of all healthy and unhealthy foods included in the prime selection task can be found in Tables S2 and S3, respectively. Non-food stimuli consisted of several positive categories, such as animals, flowers, sceneries and babies. Pictures with a CC0 license have been retrieved from Pixabay and have also been edited. These are available at https://osf.io/sjcx7/.

Food choice task items and constraints for selection by the researcher(s)

Foods were selected under certain constraints for their inclusion in the food choice task. In laboratory settings, participants were offered a food item at the end of the experiment (see *Food choice task*). However, several healthy and unhealthy foods cannot be safely stored in the laboratory due to decay (e.g., fresh fruit, cooked meals). Tables S2 and S3 show whether foods are suitable for laboratory storage or not. Similarly, due to health and safety regulations, we have chosen to provide participants with 'cupboard' items which can be bought in small packets (i.e., serving size). Participants were instructed that the food they would receive would be selected by the researcher(s). It was considered that participants should not be offered foods that they had rated negatively (i.e., 'least liked') and therefore this additional constraint was placed on the selection process. At the end of the experiment, participants were shown the foods they have chosen and were asked to wait for the researcher to select the food. With a total maximum of 16 foods being chosen, a 4 $\times$ 4 $\times$ 4 $\times$ 4 grid of food names appeared on the screen and 'most liked' foods were programmed to appear on the left side of the screen (see Figure S2). This procedure would ensure that the selection of 'least liked' foods was avoided.

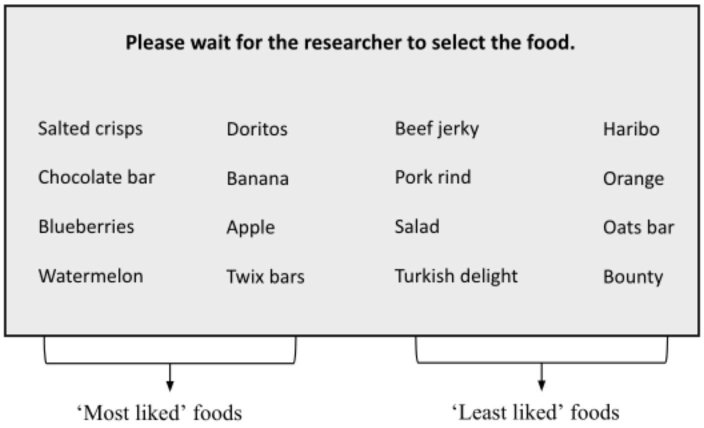

Figure S2: Schematic of food selection page for participants in laboratory settings. There was a variable number of chosen foods being presented at the end of the experiment in laboratory settings, with a maximum of 16. The names of the foods were shown in a grid as shown above. Foods presented on the left columns were 'most liked' and foods shown on the right columns were 'least liked'. The researcher(s) then selected a 'most liked' food to offer for consumption.

Table S2: Descriptive and nutritional information of healthy foods in the prime selection task

Food description		Nutritional information per 100g						Serving
Item	Lab∗	KCals	Fat	Sats	Carbs	Sugar	Salt	g
Raw almonds	Yes	587	49.00	3.70	9.50	3.90	0.00	25.0
Oats & honey bar	Yes	456	17.20	72.40	64.50	28.30	0.80	42.0
Summer berries bar	Yes	332	4.20	2.00	55.00	16.00	0.06	19.0
Chocolate & orange bar	Yes	339	5.90	2.40	52.00	18.00	0.01	19.0
Nutri-grain raisin bake	Yes	377	8.80	1.20	69.00	41.00	0.45	45.0
Beetroot & parsnip crisps	Yes	325	2.50	0.50	54.00	47.00	2.00	18.0
Pineapple crisps	Yes	344	0.00	0.00	81.00	70.00	0.00	20.0
Greek salad	No	116	9.40	2.60	4.60	3.30	0.72	185.0
Gherkin	No	20	0.20	0.08	2.60	2.60	1.23	100.0
Asparagus	No	29	0.60	0.10	2.00	1.90	0.01	62.0
Brussel sprouts	No	51	1.40	0.30	4.10	3.10	0.02	80.0
Radish	No	14	0.20	0.10	1.90	1.90	0.10	80.0
Carrots	No	42	0.30	0.10	7.90	7.40	0.00	100.0
Celery	No	10	0.20	0.10	0.90	0.90	0.15	90.0
Peppers	No	23	0.23	0.10	3.83	3.66	0.01	125.0
Beetroot	No	42	0.35	0.20	7.05	7.01	0.18	100.0
Strawberry	No	30	0.10	0.01	6.00	6.00	0.01	100.0
Orange	No	41	0.20	0.00	8.20	8.20	0.00	100.0
Grapes	No	66	0.10	0.10	15.40	15.40	0.01	100.0
Banana	No	103	0.30	0.10	23.20	20.90	0.00	150.0
Watermelon	No	33	0.30	0.10	6.90	6.90	0.01	90.0
Blueberries	No	68	0.30	0.03	14.50	10.00	0.00	100.0
Apple	No	53	0.10	0.01	11.80	11.80	0.00	133.0
Cherries	No	52	0.10	0.10	11.50	11.50	0.01	100.0
Raspberries	No	32	0.30	0.10	4.60	4.60	0.00	100.0

Note. Lab∗- suitability of foods for storage in the laboratory and offer to participants after the food choice task. Carbs: Carbohydrates; KCals: Energy in kilocalories; Fat: Total fat; Sats: Saturates; g: grams

Table S3: Descriptive and nutritional information of unhealthy foods in the prime selection task

Food description		Nutritional information per 100g						Serving
Item	Lab∗	KCals	Fat	Sats	Carbs	Sugar	Salt	g
Crisps- salted	Yes	526	31.90	2.60	51.50	0.40	1.40	25.0
Crisps- salt & vinegar	Yes	519	30.80	2.50	52.60	1.00	1.62	25.0
Crisps- cheese & onion	Yes	520	30.60	2.50	52.60	3.30	1.23	25.0
Quavers- cheese	Yes	536	30.80	2.70	62.10	2.70	2.14	16.0
Frazzles	Yes	483	22.70	1.70	62.50	2.40	2.76	18.0
Doritos- cheese	Yes	499	26.30	2.40	55.40	2.60	1.27	30.0
Cheese puffs	Yes	546	33.00	4.00	56.10	6.60	1.96	16.5
Crisps- chicken	Yes	525	30.00	2.80	54.00	2.20	1.50	27.0
Crisps- steak	Yes	526	31.00	2.80	53.00	2.30	1.50	27.0
Crisps- bacon	Yes	524	30.00	2.70	54.00	2.90	1.50	27.0
Mini cheddars	Yes	512	29.20	11.60	50.10	5.10	2.50	25.0
Corn snack- roast beef	Yes	492	25.00	2.20	59.00	3.00	1.73	22.0
Corn snack- onion	Yes	492	25.00	2.10	60.00	3.00	1.55	22.0
Beef jerky	Yes	315	3.50	1.50	32.40	20.60	3.60	40.0
Pork rind	Yes	626	46.50	17.00	1.60	0.10	2.90	22.5
Twirl bar	Yes	535	30.00	18.00	57.00	56.00	0.22	21.5
Crunchy bar	Yes	466	17.00	10.00	73.00	65.00	0.71	32.0
Chocolate bar	Yes	534	30.00	18.00	57.00	56.00	0.24	45.0
Chocolate caramel bar	Yes	484	23.00	14.00	63.00	53.00	0.37	45.0
Bounty coconut bar	Yes	487	25.70	21.20	58.90	48.20	0.25	28.5
Milk chocolate buttons	Yes	535	30.00	18.00	57.00	56.00	0.24	40.0
Aero peppermint	Yes	531	28.90	17.10	61.60	60.80	0.25	36.0
Twix bars	Yes	495	24.00	13.90	64.60	48.80	0.44	25.0
Turkish delight	Yes	363	6.70	3.80	74.00	64.00	0.36	51.0
Haribo starmix	Yes	342	0.50	0.10	7.00	47.00	0.03	50.0

Note. Lab∗- suitability of foods for storage in the laboratory and offer to participants after the food choice task. Carbs: Carbohydrates; KCals: Energy in kilocalories; Fat: Total fat; Sats: Saturates; g: grams

Follow-up study questionnaire

Please answer the following questions about the main word task you completed. Please try to respond honestly. Research shows that people, when answering questions, prefer not to pay attention and minimise their effort as much as possible. If you are reading this, please select "none of the above" on the next question.

Q1. What was this study about?

☐ Food adverts ☐ Body weight ☐ Healthy diets ☐ None of the above

Q2. Did you notice any differences about your responses on separate occasions? Please select all that apply.

☐ Faster to categorise positive words
☐ Faster to categorise negative words
☐ Faster to categorise words towards the end of the task
☐ Slower to categorise positive words
☐ Slower to categorise negative words
☐ No differences observed

Q3. How frequently did you see the content of the picture that was presented before the word?

1=Never; 2=Very infrequently; 3=Somewhat infrequently; 4=Occasionally; 5=Somewhat frequently; 6=Very frequently; 7=Always

Q4. Please indicate whether you believe that the picture content influenced your responses in any way by selecting all statements below that apply to your performance in the word task.

☐ Faster to categorise positive words when the picture was positive (i.e., picture you liked the most)
☐ Faster to categorise negative words when the picture was negative (i.e., picture you liked the least)
☐ Slower to categorise positive words when the picture was negative
☐ Slower to categorise negative words when the picture was positive
☐ Responses were not influenced by the content of the pictures

Q5. Did you find all the words in the task clearly positive or negative? Certain words may be considered unclear or ambivalent. These may be words that have both positive and negative meaning for you depending on the context. If not, please type in any words in the text box
- Yes
- No [open-ended response]

Q6. Obama was the first American president.
7-point Likert scale (1=strongly disagree to 7=strongly agree)

Q7. Did you purposefully use any kind of strategy to make your responses faster and/or more accurate? Please select all that apply.

☐ Slowed down to be more accurate
☐ Responded fast most of the time and ignored any errors
☐ No strategy used
☐ Other [open-ended response]

Q8. How many times were you interrupted during the word task (e.g., by the phone ringing or by somebody trying to talk to you)?
☐ 0 ☐ 1 ☐ 2 ☐ 3 ☐ 4 ☐ Other [numerical input up to two digits]

Explanation of attention and instruction manipulation checks

As described in Kees, Berry, Burton, & Sheehan (2017), we have added one question in the survey to serve as an attention check (Q6). For this question, participants should respond with 1=strongly disagree if they are paying attention. We have also included a modified instructional manipulation check (IMC) that fits the research context of the study, whereby participants should read the survey instructions carefully to correctly answer Q1. The survey also includes an adapted question from (Waters & Li, 2008), that is Q8, which aims to capture how many times participants were interrupted during the affective priming paradigm ("word task").

Additional details about the analysis plan

Pre-processing of the data and confirmatory analyses were exclusively conducted in R (R Core Team, 2017) via RStudio (RStudio Team, 2016) and all scripts are available on the Open Science Framework (OSF; https://osf.io/73xfr). For Bayesian t-tests and correlations the "BayesFactor" package (Morey & Rouder, 2018) was used and for the reported frequentist tests the "jmv" (jamovi) package (Selker, Love, & Dropmann, 2018) was employed.

Although statistical tests were not conducted for performance in the FCT alone, such as reaction time differences between trial types, we would report descriptively the probabilities of choosing most liked food items in the most liked vs least liked trials when both healthy and unhealthy food pairs were presented, which should be above 0.5 if prime selection according to liking was successful (also see Veling et al., 2017).

For all t-tests (H1-H3), Cohen's d_{av} was reported, which uses the average standard deviation of both measures in a paired-samples comparison and can be similar to Cohen's d_s effect size for between-subject designs, increasing its utility for potential meta-analyses (Lakens, 2013). The formula for the calculation, as presented below, was obtained from Lakens (2013). The mean difference of the two repeated measures (mean of the difference scores) is divided by the average standard deviation of both measures. The R code has been adapted from an existing script from Anvari & Lakens (2019), available at https://github.com/Lakens/anchor_based_methods_SESOI. The script also provides code for the calculation of confidence intervals (CIs) for Cohen's d_{av}, which were also reported.

$$Cohen's \quad d_{av} = \frac{Mean \quad difference}{\sqrt{\frac{SD_1^2+SD_2^2}{2}}}$$

The Shapiro-Wilk test of normality was conducted for every t-test under H1, H2 and H3. If the normality assumption was violated under $p < 0.005$ for any of planned comparisons in a set of predictions for RTs (H1a, H2a-H2d), only the results with log-transformed values (e.g., $\text{logRT}_{\text{con}} < \text{logRT}_{\text{inc}}$ for H1a) would be reported. For example, if normality was violated for H2c under H2 (i.e., only RT difference scores for unhealthy food prime trials), we would log-transform RTs for all comparisons (H2a, H2b, H2c, H2d). For error-related predictions (H1b, H3a-H3d), Bayesian t-tests would be performed as planned. However, as part of the supplementary frequentist statistics, Wilcoxon signed rank tests would also be performed.

Robustness check results

The preregistered hypotheses of the study were tested under different aggregation and reduction conditions, as outlined below. Following the sensitivity analyses, the findings of the study would be considered "robust" if the results were consistent with those of the main preregistered analyses presented in the *Preregistered analyses* section.

1. *Error rate exclusion*: We would set a more conservative criterion for participant exclusions based on error rates, which would require error rates smaller or equal to 0.25 and all hypotheses would be re-tested (H1-H4). However, there were not enough exclusions to deem further analyses informative (only three participants would be excluded in each cohort).

2. *Mean reaction times*: In line with previous literature on affective priming effects in the food domain (Lamote, Hermans, Baeyens, & Eelen, 2004; Verhulst, Hermans, Baeyens, Spruyt, & Eelen, 2006), we tested whether H1, H2 and H4 results were consistent when individual means were used in place of individual median reaction times. Since mean RTs could be more sensitive to outliers compared to median RTs, we employed previously reported criteria for outlier removal, as listed below.

Criterion 2a. Trials with RTs shorter than 250ms were removed, as reported in Lamote et al. (2004). Exclusion of longer RTs (>1500ms) did not apply to the present APP design (MaxRT = 1500ms).

Criterion 2b. RTs that deviated more than 2.5 standard deviations from the mean of each design cell for food (congruence $\times$ healthiness $\times$ liking) and non-food prime trials (congruence) were removed (Verhulst et al., 2006).

Considering that only Bayesian statistical tests would inform the conclusions of the study, only Log(BF_{10}) values are presented in Table S4. Sensitivity analyses showed that results from preregistered tests were robust to different RT aggregation and reduction criteria. This is an important issue of replicability as it can be inferred that observed priming effects were not affected by different statistical decisions, that can often be subjective.

Table S4: Robustness check results for preregistered hypotheses from the laboratory and online cohorts

		Criterion 2a		Criterion 2b	
	Cohort	Log(BF_{10})	Evidence interpretation	Log(BF_{10})	Evidence interpretation
H1a	Laboratory	16.06	*Extreme* evidence for H_1	18.39	*Extreme* evidence for H_1
H2a	Laboratory	51.79	*Extreme* evidence for H_1	50.12	*Extreme* evidence for H_1
H2b	Laboratory	38.27	*Extreme* evidence for H_1	31.86	*Extreme* evidence for H_1
H2c	Laboratory	32.18	*Extreme* evidence for H_1	34.98	*Extreme* evidence for H_1
H2d	Laboratory	-2.51	*Strong* evidence for H_0	-2.27	*Strong* evidence for H_0
H4a	Laboratory	-2.77	*Strong* evidence for H_0	-3.24	*Strong* evidence for H_0
H4b	Laboratory	-2.71	*Strong* evidence for H_0	-2.82	*Strong* evidence for H_0
H4c	Laboratory	-1.69	*Moderate* evidence for H_0	-1.60	*Moderate* evidence for H_0
H1a	Online	21.48	*Extreme* evidence for H_1	24.78	*Extreme* evidence for H_1
H2a	Online	26.40	*Extreme* evidence for H_1	24.94	*Extreme* evidence for H_1
H2b	Online	16.55	*Extreme* evidence for H_1	15.19	*Extreme* evidence for H_1
H2c	Online	15.27	*Extreme* evidence for H_1	15.70	*Extreme* evidence for H_1
H2d	Online	-2.95	*Strong* evidence for H_0	-2.47	*Strong* evidence for H_0
H4a	Online	-1.03	*Anecdotal* evidence for H_0	-1.19	*Moderate* evidence for H_0
H4b	Online	-0.78	*Anecdotal* evidence for H_0	-1.30	*Moderate* evidence for H_0
H4c	Online	-2.79	*Strong* evidence for H_0	-2.99	*Strong* evidence for H_0

Note. Evidence is interpreted for the alternative hypothesis (H_1) compared to the null (H_0) and vice versa. All hypotheses are statistically defined in the *Preregistered analyses* section. Criteria 2a and 2b are as defined above. Log(BF10): Natural logarithm of BF_{10}

Data quality checks

The follow-up study questionnaire included an attention check question (Q6; Kees et al., 2017) and the percentage of correct responses was recorded for both laboratory and online cohorts as a data quality check. Although participants could have been paying attention to the actual questions, the follow-up questionnaire also included a modified instructional manipulation check (IMC; Q1) to examine whether they had read the instructions for the questionnaire before answering the questions. The self-reported number of times participants were interrupted during the APP was also recorded (adapted from Waters & Li, 2008), as distractions in online settings may be a potential limitation for experimental studies involving reaction time tasks. The sample sizes for the laboratory and online cohorts for this exploratory analysis were 200 and 198 respectively, as participants who did not complete the questionnaires, and were excluded from confirmatory (APP and FCT) analyses, were not included.

Although there are potential issues that can affect data quality in online studies (e.g., see Chandler, Paolacci, Peer, Mueller, & Ratliff, 2015; Paolacci & Chandler, 2014), the percentage of participants who carefully read the instructions on the follow-up study questionnaire and chose the correct answer on the IMC was slightly higher in the online cohort (M = 86.36, SD = 34.40; N = 160) than in the laboratory cohort (M = 80.00, SD = 40.10; N = 171). The percentage of participants who answered correctly on the attention check question was very high for both laboratory (M = 90.00, SD = 30.08; N = 180) and online cohorts (M = 94.95, SD = 21.95; N = 188). The percentage of participants who reported any self-reported interruptions during the APP,

irrespective of the number (e.g., one or two), was also examined and there were no considerable differences observed between the laboratory (M = 21.50, SD = 41.19; N = 43) and online cohorts (M = 24.75, SD = 43.26; N = 49). In addition to the data quality checks reported above, online testing was highly precise, as shown by the low number of exclusions based on error rates (0.019 % of participants) and the fact that added noise in the APP data, such as outliers, was not observed (e.g., see dispersion of median RTs in Figures 5 and 6).

Follow-up study questionnaire results

The follow-up study questionnaire allowed us to gather both quantitative and qualitative data regarding issues of awareness and strategic responding in the APP. Please note that validity checks were conducted for all questionnaire responses and participants who were found to provide contradicting responses were excluded from this analysis. For example, on Q7 if a participant responded that they did not use a strategy but also checked one of the other boxes for response strategies, their response was deemed invalid. This validity check was applied to Q2, Q4 and Q7. Participants who had invalid responses in any of these questions were excluded. Participants who did not complete the questionnaire and were excluded from confirmatory analyses were also not included. The final sample sizes for the results reported in Table S5 are 194 and 192 for the laboratory and online cohorts, respectively.

Table S5: Standardized results from the follow-up study questionnaire for laboratory and online cohorts

	Laboratory		Online	
	M	*SD*	*M*	*SD*
Q2. Awareness of RT differences (% selected)				
Faster to categorise positive words	40.72	49.26	23.96	42.79
Faster to categorise negative words	22.16	41.64	14.06	34.85
Faster to categorise words towards the end	22.16	41.64	21.35	41.09
Slower to categorise positive words	6.70	25.07	10.42	30.63
Slower to categorise negative words	23.20	42.32	15.10	35.90
No differences observed	22.68	41.98	42.71	49.59
Q3. Awareness of prime content (1-7)	5.39	1.46	5.63	1.46
Q4. Awareness of affective congruence (% selected)				
Faster with positive prime-target pairs	65.46	47.67	30.21	46.04
Faster with negative prime-target pairs	42.78	49.60	14.58	35.39
Slower with negative prime-positive target pairs	34.02	47.50	17.71	38.27
Slower with positive prime-negative target pairs	43.30	49.68	20.83	40.72
Responses were not influenced by prime content	20.10	40.18	53.12	50.03
Q5. Ambivalence of targets (% Yes ∗)	70.62	45.67	62.50	48.54
Q7. Response strategies (% selected)				
Slowed down to be more accurate	19.07	39.39	10.42	30.63
Responded consistently fast, ignoring errors	28.87	45.43	17.71	38.27
No strategy used	52.06	50.09	72.40	44.82

Note. For checkbox questions (Q2, Q4, Q7), participants could select all answers that apply, such as for awareness of affective congruence they could notice they were faster with positive prime-target pairs as well as slower with positive prime-negative target pairs. Awareness of prime contentL 1="Never", 4="Occasionally", 7="Always"; * Yes- found all words clearly positive or negative

The questionnaire items and results have been categorised into the following themes: awareness of RT differences (Q2), awareness of prime content (Q3), awareness of affective congruence (Q4), ambivalence of targets (Q5) and response strategies (Q7). Standardised results, excluding open-ended responses, can be found in Table S5. An important question regarding these outcomes is whether the participants in the laboratory cohort had a greater awareness of the task design in general compared to participants recruited from the general population online via Prolific. The laboratory cohort primarily included undergraduate students from the School of Psychology at Cardiff University. Although experimental testing in the laboratory may have higher precision compared to online testing, which was in fact not the case in this study, it is also possible for the laboratory sample to show greater bias and demand characteristics in their responses, especially in the presence of a researcher (see Podsakoff, MacKenzie, Lee, & Podsakoff, 2003 for review).

The findings presented in Table S5 corroborate this assumption, as on average participants in the laboratory cohort were more aware of reaction time differences for types of targets and the effects of affective congruence on their performance for different trial types. For example, 53.12% of participants in the online cohort reported that they believed their responses were not influenced by the content of the pictures (i.e., primes) on Q4 , while only 20.10% of participants who completed the study in the laboratory reported their performance was not influenced by the primes. The laboratory cohort were also more aware of different trial types (affective congruence design cells) with more than 30% selecting all options on Q4. Also, in the online cohort 72.40% of participants reported that they did not purposefully use any kind of strategy to make their responses faster and/or more accurate. The ambivalence of targets which were included in the APP was another issue that was examined using self-reports and for both laboratory and online cohorts many participants indicated that some words were either positive or negative depending on context (e.g. *alone*, *brave*) and that certain words were neutral (e.g., *travel*) and less positive due to personal experiences (e.g., *party*, *hope*) or religious associations (e.g., *heaven*, *angel*).

References

Anvari, F., & Lakens, D. (2019). Using Anchor-Based Methods to Determine the Smallest Effect Size of Interest [Preprint]. *PsyArXiv*. https://doi.org/10.31234/osf.io/syp5a

Blechert, J. (2019). Food-Pics_Extended—An Image Database for Experimental Research on Eating and Appetite: Additional Images, Normative Ratings and an Updated Review. *Frontiers in Psychology*, *10*(307), 1–9.

Blechert, J., Meule, A., Busch, N. A., & Ohla, K. (2014). Food-pics: An image database for experimental research on eating and appetite. *Frontiers in Psychology*, *5*(617), 1–10. https://doi.org/10.3389/fpsyg.2014.00617

Chandler, J., Paolacci, G., Peer, E., Mueller, P., & Ratliff, K. A. (2015). Using Nonnaive Participants Can Reduce Effect Sizes. *Psychological Science*, *26*(7), 1131–1139. https://doi.org/10.1177/0956797615585115

Grühn, D. (2016). An English Word Database of EMOtional TErms (EMOTE). *Psychological Reports*, *119*(1), 290–308. https://doi.org/10.1177/0033294116658474

Kees, J., Berry, C., Burton, S., & Sheehan, K. (2017). An analysis of data quality: Professional panels, student subject pools, and amazon's mechanical turk. *Journal of Advertising*, *46*(1), 141–155. https:

//doi.org/10.1080/00913367.2016.1269304

Lakens, D. (2013). Calculating and reporting effect sizes to facilitate cumulative science: A practical primer for t-tests and ANOVAs. *Frontiers in Psychology*, *4*. https://doi.org/10.3389/fpsyg.2013.00863

Lamote, S., Hermans, D., Baeyens, F., & Eelen, P. (2004). An exploration of affective priming as an indirect measure of food attitudes. *Appetite*, *42*(3), 279–286. https://doi.org/10.1016/j.appet.2003.11.009

Morey, R. D., & Rouder, J. N. (2018). *BayesFactor: Computation of bayes factors for common designs*. Retrieved from https://CRAN.R-project.org/package=BayesFactor

Paolacci, G., & Chandler, J. (2014). Inside the Turk: Understanding Mechanical Turk as a Participant Pool. *Current Directions in Psychological Science*, *23*(3), 184–188. https://doi.org/10.1177/0963721414531598

Podsakoff, P. M., MacKenzie, S. B., Lee, J.-Y., & Podsakoff, N. P. (2003). Common method biases in behavioral research: A critical review of the literature and recommended remedies. *Journal of Applied Psychology*, *88*(5), 879–903. https://doi.org/10.1037/0021-9010.88.5.879

R Core Team. (2017). *R: A language and environment for statistical computing*. Retrieved from https://www.R-project.org/

RStudio Team. (2016). *RStudio: Integrated development environment for r*. Retrieved from http://www.rstudio.com/

Selker, R., Love, J., & Dropmann, D. (2018). *Jmv: The "jamovi" analyses*. Retrieved from https://CRAN.R-project.org/package=jmv

Veling, H., Chen, Z., Tombrock, M. C., M. Verpaalen, I. a., Schmitz, L. I., Dijksterhuis, A., & Holland, R. W. (2017). Training Impulsive Choices for Healthy and Sustainable Food. *Journal of Experimental Psychology: Applied*, *23*(1), 1–14. https://doi.org/10.1037/xap0000112

Verhulst, F., Hermans, D., Baeyens, F., Spruyt, A., & Eelen, P. (2006). Determinants and predictive validity of direct and indirect measures of recently acquired food attitudes. *Appetite*, *46*(2), 137–143. https://doi.org/10.1016/j.appet.2005.11.004

Waters, A. J., & Li, Y. (2008). Evaluating the utility of administering a reaction time task in an ecological momentary assessment study. *Psychopharmacology*, *197*(1), 25–35. https://doi.org/10.1007/s00213-007-1006-6
